# Autism: epidemiology, genetics, molecular mechanisms, clinical features, diagnosis, and management

**DOI:** 10.1186/s43556-026-00613-2

**Published:** 2026-10-20

**Authors:** Ruonan Fan, Yilin Wang, Tingting Miao

**Affiliations:** Department of Human Anatomy, School of Basic Medicine, China Medical University, Shenyang, 110122 China

**Keywords:** ASD, Epidemiology, Genetic mechanism, Molecular pathology, Animal models, Precise diagnosis

## Abstract

Autism spectrum disorder (ASD) is a lifelong neurodevelopmental condition characterized by persistent deficits in social communication and restricted, repetitive behaviors. Global prevalence has risen steadily, yet reported rates vary markedly across regions and populations. These discrepancies reflect inequities in diagnostic access and screening capacity rather than true differences in incidence. This review integrates recent advances across the full ASD research spectrum. We first examine global epidemiological patterns and demographic disparities. We then dissect the genetic architecture of ASD, including rare and common variants, copy number variations, and gene–environment interactions that disrupt prenatal and early postnatal brain development. At the molecular level, diverse risk factors converge on shared neurodevelopmental mechanisms. These include synaptic dysfunction, dysregulated signaling through mTOR, Ras-ERK-MAPK, Wnt and Notch pathways, impaired neurogenesis, chronic neuroimmune inflammation, and excitatory–inhibitory imbalance. We also discuss how these mechanisms manifest in core clinical features, including social deficits, repetitive behaviors, and sensory abnormalities. We summarize current diagnostic standards and their limitations. Cross-species animal models, from *Drosophila* to non-human primates, are evaluated for their essential roles in mechanistic validation and therapeutic screening. Finally, we assess clinical management strategies, ranging from behavioral and educational interventions to pharmacological and supportive services. By tracing a continuous path from risk factors at the population level through molecular pathophysiology to interventions guided by mechanism, this review offers an integrative synthesis that connects basic discovery to clinical translation. We further discuss how multi-omics and artificial intelligence may advance precise diagnosis rooted in mechanism and personalized treatment.

## Introduction

ASD is a neurodevelopmental condition with considerable clinical variation, characterized by persistent difficulties in social communication and interaction alongside restricted interests and repetitive behaviors. In recent years, global prevalence has risen steadily, reflecting broader diagnostic criteria, expanded screening programs, and growing public awareness. However, much of this increase reflects differences in how countries define, diagnose, and report the condition rather than a true rise in incidence. Epidemiological studies reveal marked geographic and socioeconomic disparities: prevalence tends to be higher in North America, wealthy Asia–Pacific countries, and Europe, whereas South Asia, Africa, and other low and middle income regions report lower rates due to limited healthcare access and widespread underdiagnosis [1]. China follows a similar pattern, with reported prevalence remaining below the global average and notable gaps in early screening and standardized care [2]. These trends place significant strain on families and health systems worldwide.

Behind these epidemiological differences, the causes of ASD are complex and multifactorial, arising from interactions between genetic susceptibility and environmental triggers. Heritability estimates range from 70% to 90% [3]. With rare, common, and non-coding variants all contributing to the disruption of core neurodevelopmental processes such as neuronal migration and synapse formation [4, 5]. Environmental factors, including adverse perinatal events, can alter epigenetic marks and amplify genetic risk [6]. These gene–environment interactions converge at the molecular and cellular level, producing defects in synaptic function, abnormal signaling through mTOR, Wnt, and Notch pathways, chronic neuroinflammation, and loss of excitatory–inhibitory balance [7–12]. To systematically validate these molecular mechanisms and explore therapeutic targets, reliable disease models have become essential. Animal models, including rodents, *Drosophila*, zebrafish, and non-human primates, each offer distinct advantages, and their combined use helps capture different aspects of disease heterogeneity that no single system can provide [13]. Nevertheless, biological insights from these models have yet to be effectively translated into clinical breakthroughs. Diagnosis still depends on behavioral criteria from the DSM-5-TR and ICD-11, with no biomarkers in routine use. Rehabilitation services remain unevenly distributed, and findings across genetics, neuroscience, and clinical science are fragmented, leaving an integrated view that connects molecular mechanisms to patient care largely missing [14].

Many existing reviews discuss genetic variants, molecular pathways, or animal models in isolation, with few tracings how mechanistic findings move systematically from bench to bedside. Herein, we advance an integrative Mechanistic Convergence and Translational Continuity (MCTC) Framework for understanding ASD. This framework posits that diverse genetic and environmental risk factors converge on a limited set of conserved neurodevelopmental mechanisms, synaptic dysfunction, signaling pathway dysregulation (mTOR, Ras-ERK-MAPK, Wnt, and Notch), and excitation–inhibition (E/I) imbalance, thereby creating a rational bridge from basic discovery to precision intervention. We examine this convergence across four interlinked themes: (1) how disease-causing variants disrupt shared neurodevelopmental pathways; (2) how environmental factors alter epigenetics and interact with genetic risk; (3) how animal models validate these mechanisms and enable therapeutic screening; and (4) the clinical potential and barriers of multi-omics and artificial intelligence. We also address epidemiology, clinical characteristics, and intervention strategies. By foregrounding mechanistic convergence and translational continuity across these domains, we aim to provide a focused, conceptually driven synthesis rather than a broad overview, and to identify priorities for future research.

## Epidemiological features and diagnostic standards of ASD

This section turns first to ASD epidemiology, examining global and regional variations in prevalence, demographic patterns, and risk factors. Drawing on data from Global Burden of Disease (GBD) 2021, the World Health Organization (WHO), and the Centers for Disease Control and Prevention (CDC), with diagnoses based on DSM-5-TR criteria, we outline the current prevalence picture and examine the main drivers of regional differences.

### Global prevalence and regional distribution patterns

The GBD 2021 estimates global ASD prevalence at roughly 61.8 million people (95% uncertainty interval: 52.1–72.7 million) [1]. A concurrent WHO estimate suggests a similar total about 62 million (1 in 127 people) [15]. Yet these global figures mask wide regional variation. Reported levels track closely with economic development, healthcare resources, and diagnostic capacity. Higher-income countries generally record higher proportions; East Asia, North America, and Western Europe rank among the highest globally, while South Asia, Africa, and other lower-income regions fall at the lower end [16].

The Asia–Pacific region illustrates this divide clearly. High-income economies there, Japan, South Korea, and Singapore, show the highest reported rates, with age-standardized prevalence reaching 1,559.5 per 100,000. Japan alone leads the world at 1,586.9 per 100,000 [17]. China, the region’s most populous country, reported an age-standardized prevalence of 655.7 per 100,000 in 2021, totaling roughly 3.12 million cases, somewhat below the global average [18]. Yet limited screening and weak diagnostic capacity at the primary-care level mean many cases are likely missed, and the true prevalence may be underestimated [2]. By contrast, low-income countries such as Bangladesh have the lowest reported rates, at 588.2 per 100,000, and rank among the lowest globally [19].

The Americas show the same north–south split, again highlighting how diagnostic capacity shapes report prevalence. In North America, reported ASD prevalence continues to rise. CDC data from 2022 (published in 2025) show 3.22% of American 8-year-olds (about 1 in 31) identified as having ASD, nearly a five-fold increase from the 0.67% (1 in 150) recorded in 2000 [20]. The data also reveal racial disparities, with Asian and Black children identified at higher rates than non-Hispanic White children [21]. Latin America, by contrast, reports much lower overall levels; age-standardized prevalence in tropical Latin America is 614.5 per 100,000. Yet national surveys in Brazil reveal an important exception. While ASD affects 1.2% of the general population, prevalence rises to 2.6% among children aged 5 to 9 and 3.8% among boys in the same age group [22], suggesting that even in regions with limited diagnostic resources, specific subgroups can show markedly elevated rates.

For Europe, the picture is one of transition: strong diagnostic capacity, moderate prevalence, and a gradual upward trend. The continental average is roughly 0.5%, with modest regional variation. Some high-income countries now approach North American levels [23]. Standardized diagnostic systems and screening tools make European data particularly reliable. Meanwhile, broader community networks and better recognition of milder cases continue to push overall prevalence slowly upward [24].

Africa and Oceania present a sharp contrast: one data scarce, the other heavily burdened. In Africa, research capacity remains limited and reliable data are few. Current models estimate continental prevalence at roughly 1%, yet weak healthcare infrastructure and a shortage of trained specialists mean many mild or hidden cases go undiagnosed, and the true burden likely exceeds current estimates [25]. Nigeria, the continent’s most populous country, reported roughly 830,000 ASD cases in 2021, the fifth-largest national caseload worldwide. Even so, its age-standardized prevalence remains well below that of high-income Western countries. This gap suggests that limited access to diagnosis, rather than true differences in incidence, is the main driver. Australia, by contrast, reports a much higher burden, with overall prevalence at about 1.7%, placing it among the highest globally [26].

Overall, the global pattern of ASD is driven mainly by detection and reporting capacity, not by true underlying incidence. Reported rates are higher in high-income regions largely because of wider diagnostic criteria, expanded screening coverage, and greater public awareness. North America, high-income Asia–Pacific countries, and Western Europe form the highest-prevalence zone, while Latin America, South Asia, and Africa face widespread underdiagnosis and underreporting due to limited clinical resources. China falls below the global average in overall reported prevalence, yet gaps remain in early identification, standardized screening, and intervention systems. Understanding this regional heterogeneity, and the diagnostic and reporting mechanisms behind it, is essential for identifying risk factors, designing targeted prevention strategies, and allocating public health resources effectively.

### Demographic characteristics of the population

With global regional patterns established, we now examine how ASD distributes across age, sex, race, and socioeconomic status. These demographic layers reveal not only who is affected, but also who is identified and who remains undiagnosed.

Children and adolescents still account for the majority of cases, though adults are increasingly recognized as significantly affected. CDC data show a male-to-female identification ratio of 3.4:1 among 8-year-olds, a gap that has narrowed but remains wide [27]. Behind this ratio lies a skew in diagnostic timing: boys are consistently identified earlier, while girls face marked delays. Over a quarter of women receive their diagnosis in adulthood only, largely because their symptoms are less obvious and frequently overlooked [28]. For adults overall, epidemiological data remain sparse, and the long-term care needs are poorly understood.

Sex differences are the most pronounced demographic feature of ASD, and their significance extends well beyond reported figures. Males are affected at higher rates than females globally, though this gap has steadily narrowed. Standardized data from 2021 report a male-to-female ratio at roughly 2.1:1, down markedly from the 4:1 reported in earlier studies, largely reflecting improved recognition of female cases [15, 29]. Among young and middle-aged adults, men still account for the greater share of cases and related needs, suggesting that the overall sex pattern has settled once recognition biases are taken into account [30]. This excess of males in the statistics largely reflects who gets identified, not who is truly affected. The same logic becomes even clearer when examing race.

At the level of race, biological susceptibility appears comparable across groups; observed variation in reported rates stems instead from unequal healthcare access, differences in health literacy, and uneven diagnostic availability, all of which shift dynamically over time [31]. National surveys in Brazil confirm this interpretation, showing that ethnic disparities in diagnosis closely track the uneven distribution of medical resources rather than any intrinsic variation in incidence [22]. In other words, race matters for ASD statistics mainly because it shapes who can reach a clinic, not because it alters biological risk. This mechanism overlaps substantially with how socioeconomic status operates.

Income and education work through similar channels. Historically, higher-income groups accounted for a greater share of diagnoses, but as public health services have expanded, this gap has gradually narrowed. Social conditions influence ASD outcomes indirectly via prenatal environment, early caregiving, and timely access to specialist services, rather than through altering biological risk itself. Taken together, disparities across age, sex, race, and socioeconomic status largely reflect differences in screening intensity, clinical capacity, and public awareness, not variation in innate susceptibility. As early detection systems improve worldwide, previously overlooked groups (girls, adults, and underserved populations) will increasingly enter the statistical record, bringing reported prevalence closer to the true underlying incidence.

### Risk factors

ASD shows marked variation across individuals and regions, driven by genetic background, perinatal stress, and environmental exposures. The sections below examine how each factor acts alone or in combination to disrupt neurodevelopment.

Genetic susceptibility sets the foundation. Twin studies, family investigations, and molecular analyses consistently place ASD heritability at 70% to 90%, with strong familial clustering [32]. Hundreds of risk genes have been identified, most operating through three functional modules [33]: regulation of embryonic brain development and neuronal migration; synapse formation and plasticity; and chromatin remodeling with transcriptional control. Disruption in any module can impair neurodevelopment. Chromosomal anomalies such as 15q11–q13 variants and 22q13.3 deletions also substantially increased risk, and relatives of affected individuals carry far higher odds than the general population [34]. In short, genetic background largely determines who is most vulnerable.

Yet genetic vulnerability alone does not dictate whether disease occurs. Adverse events during the perinatal period and early infancy cannot cause ASD by themselves, but they can compromise neural development and worsen clinical expression, effectively modulating risk [35]. These stressors fall into three broad categories: acute brain injury from birth complications such as hypoxia, asphyxia, or birth trauma; growth impediments including severe infection, malnutrition, or micronutrient deficiency in infancy; and caregiving deficits such as insufficient social interaction or environmental deprivation, which impair the maturation of social and emotional regulatory circuits. Each act on a distinct neurodevelopmental target, serving as an important secondary influence on severity.

Environmental factors act even earlier, primarily during the critical windows of fetal and early postnatal brain development. Unlike perinatal stress, environmental exposures can independently impair neurodevelopment, with prenatal exposure being the dominant source [36]. Specific risks include substance abuse and alcohol or tobacco use during pregnancy, which directly inhibit neuronal proliferation and differentiation; heavy metals and air pollutants that cross the placental barrier and comprimise fetal brain development; maternal conditions such as gestational diabetes, hypertension, autoimmune disease, or viral infection, which trigger inflammatory responses that indirectly damage fetal neural tissue; and additional factors including nutritional imbalance, severe emotional stress, or abnormal gestational duration, each exerting negative effects through distinct pathways.

**Fig. 1:**
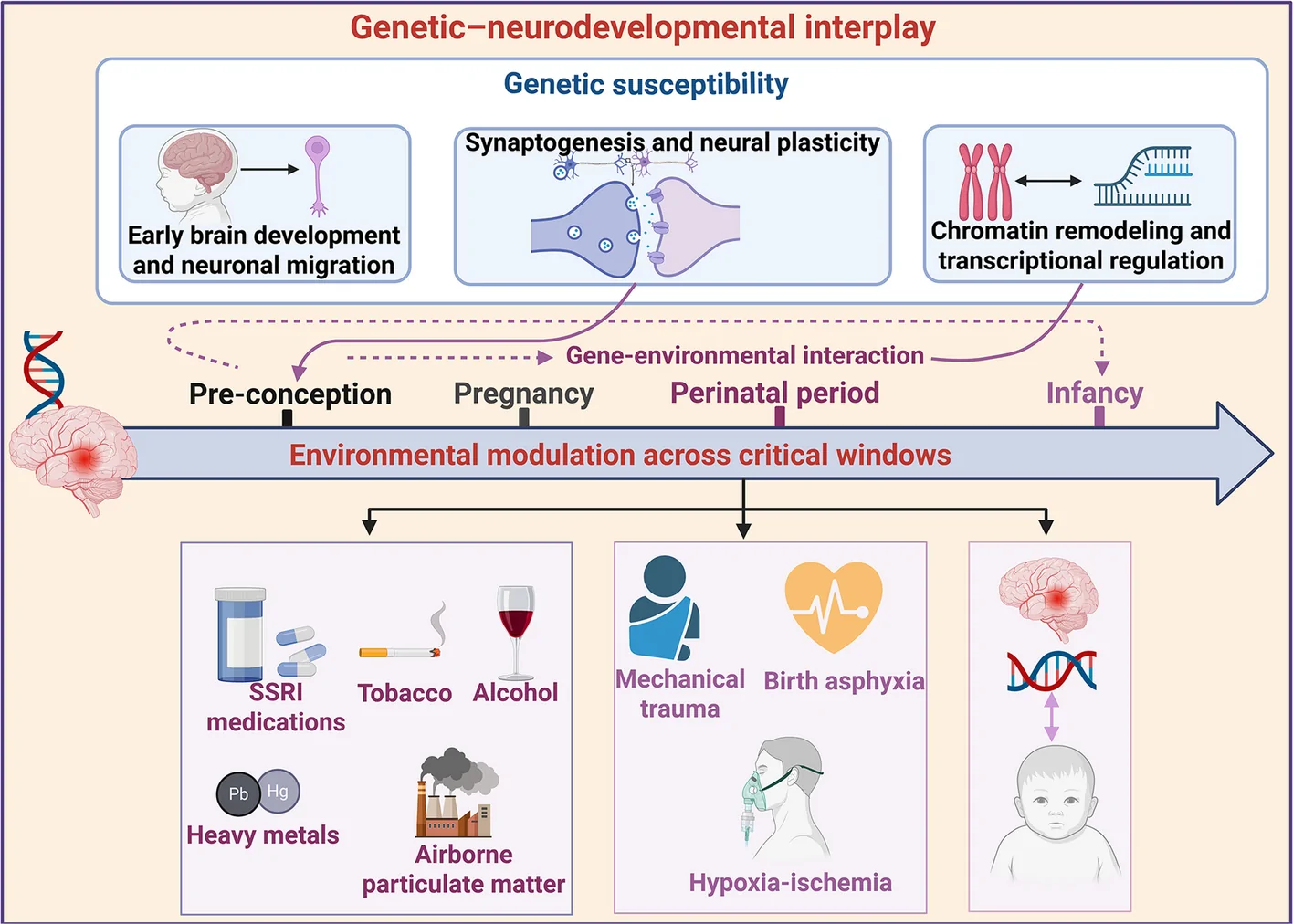
Multilevel risk factors and pathogenesis of ASD, illustrating the interplay between genetic susceptibility and environmental modulation across critical developmental stages. Integrated framework of genetic and environmental risk factors in ASD. Genetic susceptibility operates through three functional modules (early brain development and neuronal migration; synaptogenesis and neural plasticity; chromatin remodeling and transcriptional regulation), which interact with stage-specific environmental exposures across pre-conception, pregnancy, perinatal, and infant stages. Created in BioRender.com. SSRI Selective serotonin reuptake inhibitor

Together, these three factors form a layered system: genetic background provides the baseline susceptibility, while perinatal stress and environmental exposures modulate risk and shape clinical presentation thereafter. Their targets differ, yet all converge on neurodevelopment as the common final pathway. Clarifying independent contributions and interactions among them is essential for identifying high-risk groups and designing tiered prevention strategies (Fig. 1).

### ASD diagnosis: standard practice and new developments

ASD affects roughly 0.62% to 1.7% of the global population, yet delayed diagnosis remains widespread and the average age of diagnosis worldwide is still 3.5–4 years, well beyond the critical early intervention window from birth to age 3 [23, 37]. Clinicians currently rely solely on behavioral phenotyping, with no specific biomarkers available for routine use. Marked genetic and phenotypic heterogeneity, together with high rates of comorbidity, further complicate accurate identification [38].

The diagnostic landscape is anchored by two dominant nosological systems, DSM-5-TR and ICD-11 (both released in 2022), which together provide a unified clinical structure [39, 40]. However, neither system reaches the biological roots of the condition; both depend fundamentally on behavioral presentation. This behavioral paradigm creates persistent gaps between diagnostic criteria and their practical implementation, particularly in resource-constrained environments [41].

#### Diagnostic criteria

##### DSM-5-TR criteria

DSM-5-TR defines ASD through two core symptom domains, each with specific behavioral manifestations that must be present during early developmental periods and cause clinically significant impairment in social, occupational, or other important areas of functioning.

The first domain encompasses persistent deficits in social communication and social interaction across multiple contexts. These deficits manifest in three distinct areas: deficits in social-emotional reciprocity, ranging from abnormal social approach and failure of normal back-and-forth conversation to reduced sharing of interests, emotions, or affect and failure to initiate or respond to social interactions; deficits in nonverbal communicative behaviors used for social interaction, including poorly integrated verbal and nonverbal communication, abnormalities in eye contact and body language, deficits in understanding and use of gestures, and a total lack of facial expressions and nonverbal communication; and deficits in developing, maintaining, and understanding relationships, ranging from difficulties adjusting behavior to suit various social contexts to difficulties in sharing imaginative play or in making friends and absence of interest in peers.

The second domain comprises restricted, repetitive patterns of behavior, interests, or activities, manifested by at least two of the following: stereotyped or repetitive motor movements, use of objects, or speech; insistence on sameness, inflexible adherence to routines, or ritualized patterns of verbal or nonverbal behavior; highly restricted, fixated interests that are abnormal in intensity or focus, and hyper- or hyporeactivity to sensory input or unusual interest in sensory aspects of the environment [42].

DSM-5-TR eliminated traditional subtypes in favor of a single ASD diagnosis and introduced severity levels based on the degree of support required. Level 1 ("requiring support") indicates noticeable impairments in social communication and restricted, repetitive behaviors that interfere with functioning in one or more contexts. Level 2 ("requiring substantial support") applies to those with marked deficits in verbal and nonverbal social communication skills, limited initiation of social interaction, and reduced or abnormal responses to social overtures, with restricted, repetitive behaviors obvious to the casual observer. Level 3 ("requiring very substantial support") describes individuals with severe deficits causing severe impairments in functioning, very limited initiation of social interactions, and minimal response to social overtures, with restricted, repetitive behaviors markedly interfering with functioning in all spheres [43]. Its strong standardization makes it well suited to research and epidemiology [44, 45].

##### ICD-11 criteria

ICD-11 classifies ASD within the category of neurodevelopmental disorders (code 6A02) and provides diagnostic guidance that emphasizes developmental timing, functional impairment, and clinical flexibility. The ICD-11 definition requires that characteristic features of ASD manifest during the developmental period, typically in early childhood, though they may not become fully manifest until social demands exceed limited capacities or may be masked by learned strategies later in life.

ICD-11 specifies two core characteristic symptom patterns: persistent deficits in the initiation and maintenance of reciprocal social interaction and social communication, and a range of restricted, repetitive, and inflexible patterns of behavior, interests, or activities. The social communication domain highlights qualitative impairments in reciprocal social interaction, including abnormalities in the use of multiple nonverbal behaviors such as eye-to-eye gaze, facial expression, body postures, and gestures to regulate social interaction; failure to develop peer relationships appropriate to developmental level; and lack of spontaneous seeking to share enjoyment, interests, or achievements with other people [46].

A distinctive feature of ICD-11 is its explicit inclusion of diagnostic criteria for adults, acknowledging that ASD characteristics may persist throughout life and that some individuals, particularly those with higher intellectual functioning or those whose symptoms were masked during childhood, may not receive diagnosis until adulthood [47]. ICD-11 also provides clearer guidance on distinguishing ASD from intellectual developmental disorder when both conditions co-occur, emphasizing that the social communication deficits in ASD are not simply a consequence of intellectual impairment. It offers flexibility for primary-care settings and can distinguish ASD from intellectual disability comorbidity, complementing DSM-5-TR [48].

##### Comparative considerations

While DSM-5-TR and ICD-11 share substantial conceptual overlap in their core symptom definitions, important differences in emphasis and application exist. DSM-5-TR places greater emphasis on dimensional severity specification, providing clinicians with a framework for communicating the level of support needed. ICD-11, conversely, offers more flexibility in diagnostic formulation for primary care and global health settings, where specialized assessment resources may be limited.

Both systems require that symptoms cause clinically significant impairment in social, occupational, or other important areas of current functioning, and both specify that these disturbances are not better explained by intellectual developmental disorder or global developmental delay. However, DSM-5-TR allows co-diagnosis of ASD and intellectual disability, specifying the level of intellectual impairment alongside ASD severity, whereas ICD-11 provides more detailed guidance on differential diagnostic considerations when intellectual disability is present [49].

In clinical practice, the choice between diagnostic frameworks often depends on the healthcare setting, regional regulatory requirements, and the purpose of diagnosis. Research settings and epidemiological studies frequently prefer DSM-5-TR for its standardized severity metrics, while clinical services in many countries adopt ICD-11 for reimbursement and health statistics purposes. The convergence of these two major classification systems represents significant progress toward global diagnostic harmonization, yet both remain fundamentally behavioral in their approach, relying on skilled clinical observation and structured developmental history rather than biological markers [47].

#### Early detection

Core symptoms of ASD often emerge between 12 and 24 months of age, yet the average age of diagnosis worldwide remains 3.5 to 4 years, causing many children to miss the critical early intervention window from birth to age 3. Screening tools have been developed to reduce subjective bias and improve early detection, and they are widely used for community screening and follow-up of at-risk children [50, 51].

In practice, however, these tools face significant constraints. The subtle nature of atypical presentations in infancy, combined with limited awareness among frontline practitioners and uneven distribution of medical resources, contributes substantially to diagnostic delays that compromise long-term outcomes [52]. Additionally, both overdiagnosis and underdiagnosis occur in clinical practice, particularly in populations with ambiguous presentations [53].

Because current screening relies entirely on behavioral phenotyping with no specific biomarkers available for routine use, early detection remains inherently limited. Marked genetic and phenotypic heterogeneity means that early behavioral markers vary widely across individuals, and the absence of objective anchors leaves clinicians dependent on observational thresholds that may miss higher-functioning individuals, females, and those with complex presentations [38, 52].

#### Comprehensive diagnostic assessment

Within the behavioral diagnostic framework, clinicians have developed a relatively standardized assessment workflow centered on behavioral observation, rating scales, and developmental history. The operationalization of DSM-5-TR and ICD-11 criteria through standardized assessment instruments, including the Autism Diagnostic Observation Schedule, Second Edition (ADOS-2) and the Autism Diagnostic Interview-Revised (ADI-R), has improved diagnostic reliability across settings. However, these comprehensive tools require substantial training and time investment, limiting their availability in resource-constrained environments. Consequently, the gap between diagnostic criteria as specified in classification manuals and their practical implementation in diverse clinical contexts remains a persistent challenge, particularly in regions with limited access to specialized autism services [41].

A more fundamental limitation of comprehensive behavioral assessment is that the multidimensional heterogeneity of ASD means behavioral evaluation cannot distinguish underlying biological differences. Novel subtyping research has yet to reach the clinic, leaving clinicians without mechanistic frameworks to explain the diverse phenotypic presentations they observe [51]. Thus, while comprehensive assessment improves diagnostic reliability, it remains descriptive rather than explanatory.

#### Differential diagnosis

The marked genetic and phenotypic heterogeneity of ASD, together with high rates of comorbidity, substantially complicates accurate differential diagnosis [38]. Because both DSM-5-TR and ICD-11 depend on behavioral presentation, they frequently miss higher-functioning individuals, females, and those with complex comorbidities whose symptoms may be masked or attributed to other conditions [52].

Both diagnostic systems require that symptoms cause clinically significant impairment and specify that these disturbances are not better explained by intellectual developmental disorder or global developmental delay. DSM-5-TR allows co-diagnosis of ASD and intellectual disability, specifying the level of intellectual impairment alongside ASD severity. ICD-11 provides more detailed guidance on distinguishing ASD from intellectual developmental disorder when both conditions co-occur, emphasizing that the social communication deficits in ASD are not simply a consequence of intellectual impairment [49].

The lack of integration between current diagnostic criteria and emerging biological subtyping research further limits differential diagnostic precision. Without biological anchors, clinicians struggle to disentangle ASD from conditions with overlapping behavioral features such as language disorders, social communication disorder, and intellectual disability, particularly when these conditions co-occur.

#### Emerging diagnostic approaches

The limitations of the current behavioral framework, including heterogeneity, diagnostic delay, lack of objective indicators, and incomplete subtyping, have catalyzed the development of novel diagnostic paradigms. Artificial intelligence-assisted behavioral analysis, multi-omics biomarker discovery, and biological subtyping research are now opening new paths beyond traditional barriers.

More fundamentally, no highly specific biomarker is available for routine clinical use: genetic testing explains only 10% to 20% of cases, and techniques such as neuroimaging remain impractical due to cost and standardization barriers, leaving clinicians without objective diagnostic anchors [54]. Emerging approaches aim to address this gap by identifying objective biological signatures that could complement or eventually supplement behavioral criteria.

Diagnosis is expected to shift from subjective observation toward integrated behavioral and objective markers, moving toward earlier, more objective, and more precise identification. Future development of combined diagnostic systems and optimized tiered-care models should improve early recognition and precision classification, providing an evidence base for standardized intervention and better long-term outcomes. These emerging approaches represent a necessary evolution beyond the current behavioral evaluation framework, toward a model that can accommodate the biological complexity underlying ASD's diverse clinical presentations.

## Genetic research on ASD

ASD arises from the interplay of genetic, environmental, and epigenetic factors. Genetics is the primary driver, and decades of twin and family studies, together with molecular genetic analyses, have firmly established its high heritability. Rapid advances in genomics are now elucidating the genetic architecture of ASD, paving the way for earlier diagnosis, deeper mechanistic understanding, and targeted interventions. This review focuses on four core areas: the genetic basis of ASD, key genetic variants and pathogenic mutations, inheritance patterns, and gene-environment interactions. We summarize recent advances, highlight ongoing debates, and outline future directions.

### Overview of genetic basis

A strong genetic component is central to ASD. Twin studies show monozygotic concordance rates of 60% to 90%, far above the 3% to 10% seen in dizygotic twins [55, 56]. This high heritability means genetics contributes more to ASD than to most other neurodevelopmental disorders [57]. Family studies add further evidence: first-degree relatives have a 10- to 20-fold higher risk, and often show broader autism phenotypes [58].

Molecular studies have built on this foundation. Genome-wide Association Studies (GWAS), Whole Exome Sequencing (WES), and Whole Genome Sequencing (WGS) have moved the field beyond single-gene models toward a complex architecture in which rare and common variants act together across many genes [11]. Hundreds of risk genes cluster in core neurodevelopmental pathways, while copy number variants (CNVs), non-coding changes, and epigenetic alterations also contribute [59]. This genetic landscape correlates with clinical severity: children with intellectual disability tend to show a larger genetic contribution to their phenotype, whereas high-functioning patients show more heterogeneity and greater environmental influence [60]. ASD also shares genetic overlap with schizophrenia, Attention Deficit Hyperactivity Disorder (ADHD), and depression, pointing to common pathways [61]. Yet roughly 30% of cases still lack an identifiable genetic cause [62].

The genetic architecture of ASD departs markedly from classical Mendelian inheritance. It is complex and highly heterogeneous, manifesting in three core features examined below: polygenic inheritance, incomplete penetrance, and genetic heterogeneity.

### Common genetic variations and gene mutations

Genetic variation in ASD is diverse and highly heterogeneous. By their frequency and effect size, these variants fall into two broad classes: rare and common. Both shape genetic susceptibility to ASD and impair normal brain development [63].

Rare variants are the main drivers of ASD. They occur in fewer than 0.1% of the population but carry large effects; they consist largely of coding mutations and copy number variants [64]. Hundreds of ASD susceptibility genes have been identified so far, including core genes that are well validated, such as *SHANK3, NRXN1, NLGN3, NLGN4,* and *CNTNAP2*, which map to key pathways for synapse formation, neuronal migration, and signal transduction [65–68]. Other candidate genes, including *MTHFR* and *VDR*, have also been implicated in metabolic and regulatory pathways. In addition to single-gene mutations, CNVs are another major class of rare variants. They account for about 10% to 15% of ASD cases and manifest as chromosomal deletions or duplications. The most common pathogenic CNVs affect regions such as 15q11-q13, 22q13.3, and 16p11.2 [64]. These CNVs often span multiple genes and impair brain development through dosage and positional effects [69]. For example, duplication of 15q11-q13 leads to overexpression of *UBE3A* and alters circuit balance; deletion of 22q13.3 removes one copy of *SHANK3* and produces classic ASD features [70].

Unlike rare variants, which often have strong effects, common variants including single nucleotide polymorphisms (SNPs) have a frequency above 1% in the general population. Each common variant alone has only a small effect on disease risk. However, when many of them accumulate, they can significantly increase disease risk, as captured by a polygenic risk score (PRS). This makes common variants a major source of population-level genetic susceptibility [71]. GWAS shows that common variants linked to ASD are enriched in pathways involved in neurodevelopment, immune regulation, and epigenetics. Many of these risk variants are shared among several neuropsychiatric conditions [72]. Large-scale meta-analyses have identified multiple genome-wide significant loci, supporting the genetic overlap among these disorders. In addition, common variants vary significantly by ancestry; their frequency and effects differ across populations, which helps explain ethnic differences in ASD prevalence [31].

Beyond protein-coding changes, non-coding variants have recently emerged as an additional layer of risk. Variants in miRNAs, lncRNAs, promoters, and enhancers can indirectly affect the expression of neurodevelopmental genes through post-transcriptional regulation [73]. For example, variants in miRNA 137 target and regulate synapse-related genes, contributing to cognitive impairment [74, 75]. Abnormal lncRNA NEAT1 interferes with chromatin remodeling and suppresses the proliferation and differentiation of neural progenitor cells [76, 77]. The discovery of these non-coding variants improves our understanding of the layered genetic architecture of ASD and expands the disease’s regulatory network.

These insights from non-coding regions have been made possible by high-throughput sequencing, which has also accelerated the functional validation of ASD candidate genes. These genes are broadly involved in chromatin modification, synaptic plasticity, cytoskeleton organization, and neural signaling. Together, they form the molecular basis of ASD pathology. The following sections examine the functional mechanisms of several well validated risk genes in these pathways.

In terms of synaptic structure and function, SHANK family proteins are key scaffolds of postsynaptic density. Among them, *SHANK3* is the most common high-risk gene for sporadic ASD [78]. It connects glutamate receptors to the cytoskeleton and helps maintain synaptic stability and signaling [79, 80]. Loss-of-function mutations in SHANK3 are a major cause of Phelan-McDermid syndrome and typical ASD, directly disrupting information transfer across neural circuits [81, 82]. *NLGN3, CNTNAP2,* and *NRXN1* are core genes for synaptic adhesion. They work together to regulate synapse recognition and maturation. Mutations in *NLGN3* disrupt the excitatory/inhibitory balance [83–85]. Variants in *CNTNAP2* interfere with neural circuit formation during embryonic development and often co-occur with epilepsy and intellectual disability [86]. Heterozygous deletion of *NRXN1* leads to presynaptic dysfunction and language impairment [87–89].

*MECP2* encodes a methyl-binding protein that can both activate and repress transcription [90]. It regulates neurodevelopmental gene expression and neuronal morphogenesis. Imbalanced *MECP2* expression leads to abnormal dendritic development [91]. Cross-species experiments confirm that *MECP2* dysfunction induces ASD-like traits [92–94]. *KDM5* regulates neuroimmune homeostasis through epigenetic modifications [95]. Lower *KDM5* expression abnormally activates central inflammatory pathways and triggers neuroimmune dysfunction [96–98].

*FMR1* is the causative gene of fragile X syndrome (FXS), the most common known monogenic cause of ASD [99]. Although a substantial proportion of individuals with FXS (particularly males) meet the diagnostic criteria for ASD, the two conditions are not equivalent: FXS is a genetically defined disorder, whereas ASD is a behaviorally diagnosed neurodevelopmental condition, and not all individuals with FXS have ASD. It encodes the FMRP protein, which controls the transport and translation of mRNAs related to synaptic plasticity. Loss of *FMR1* reduces FMRP expression, abnormally activates the mGluR5 pathway, and alters synaptic inhibition [100]. These changes ultimately lead to social deficits and repetitive behaviors [101–103].

*UBE3A* encodes an E3 ubiquitin ligase that regulates synaptic development and remodeling in a dose-dependent way. Overexpression of *UBE3A* suppresses the BMP pathway, leading to excessive synapse elimination [104]. Loss of function, in contrast, impairs normal pruning [105]. Excessive or reduced *UBE3A* activity disrupts neural homeostasis in opposite directions, corresponding to Angelman syndrome and ASD, respectively [106–108].

Other genes contribute to ASD through their roles in neural scaffolding and immunity. *ANK3* anchors ion channels and signaling receptors on the neuronal membrane and maintains nerve impulse conduction [109]. Loss of *ANK3* function causes learning and memory deficits and motor abnormalities [110–112].

**Fig. 2:**
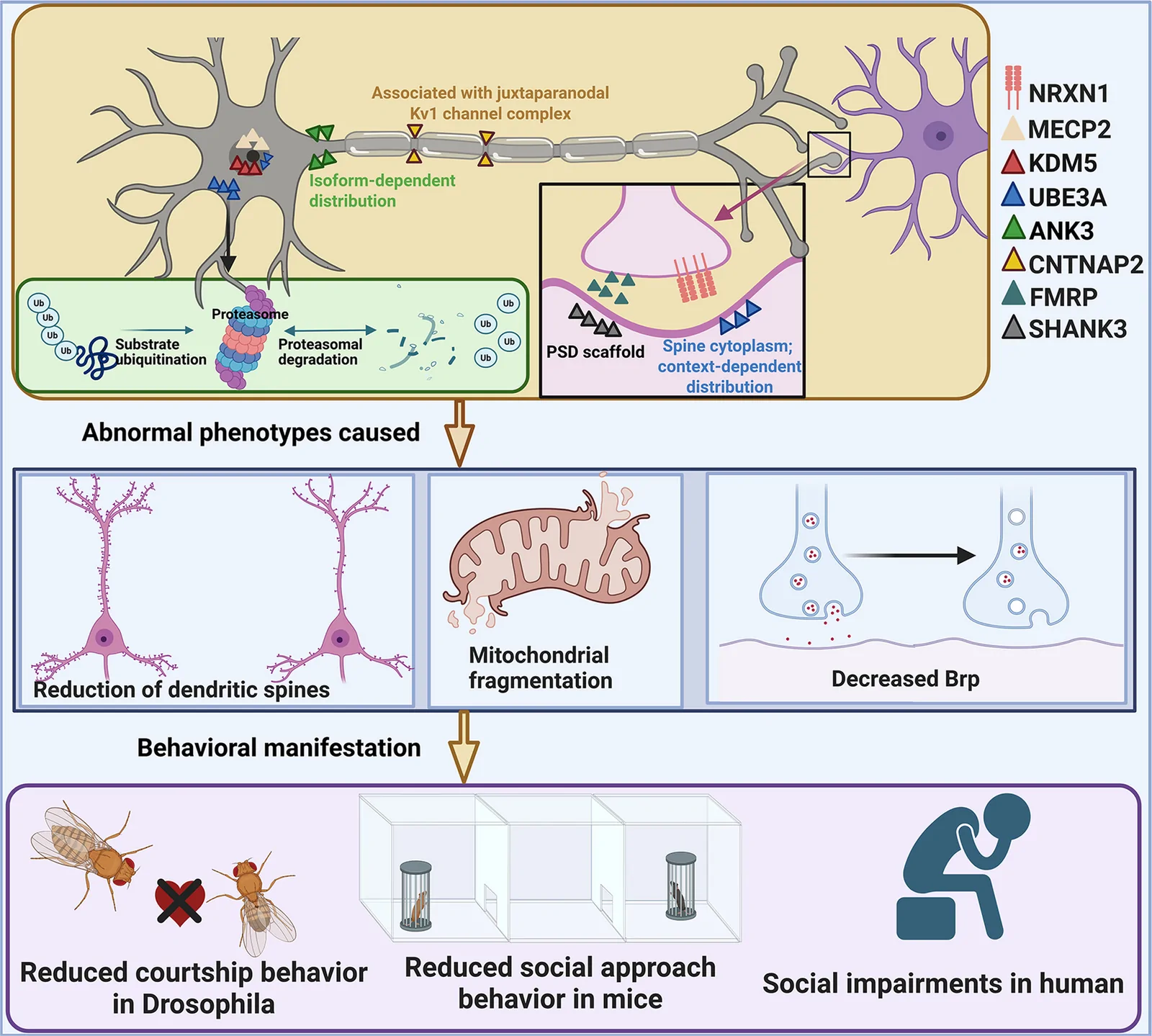
Schematic overview of cellular and behavioral pathological phenotypes induced by ASD-related genetic variants. Genetic mutations and pathway dysregulation impair key neuronal processes, such as ubiquitin–proteasome system function and synaptic vesicle regulation, leading to structural and functional abnormalities including reduced dendritic spine density, mitochondrial fragmentation, and impaired presynaptic neurotransmitter release. These cellular defects further manifest as conserved behavioral phenotypes across model organisms (e.g., reduced courtship in *Drosophila*, impaired social interaction in mice) and clinical social deficits in humans, illustrating the conserved pathological mechanisms underlying ASD pathogenesis. Graphics created with BioRender. NRXN1 Neurexin 1, MECP2 Methyl-CpG-binding protein 2, KDM5 Lysine demethylase 5, UBE3A Ubiquitin-protein ligase E3A, ANK3 Ankyrin 3, CNTNAP2 Contactin-associated protein-like 2, FMRP Fragile X mental retardation protein, SHANK3 SH3 and multiple ankyrin repeat domains 3, PSD Postsynaptic density, Brp Bruchpilot

Current ASD genetic research faces two major challenges. First, it must dissect genetic heterogeneity by integrating genomic and clinical data to refine genetic subtypes in large cohorts. Second, it must move beyond single-gene studies to understand how genes interact and how pathways cross-talk, with the goal of targeting shared neurodevelopmental mechanisms. Clinically, genetic diagnosis remains limited by high variability and incomplete penetrance. Addressing these gaps will require integrating multi-omics technologies to build models linking genetic variation, molecular expression, and clinical phenotypes, and testing whether modulating the gut-brain axis or non-coding regulatory networks can stratify patients for targeted interventions. The functional roles of these ASD-related genes are summarized in Table 1. Overall, ASD genetic research has entered a phase of multidimensional integration. Advances in technology will gradually clarify the mechanisms behind heterogeneity and support individualized diagnosis and treatment (Fig. 2).

**Table 1:** Functional summary of major ASD susceptibility genes and pathways

Gene	Core location and function	Core pathogenic mechanism in ASD	Associated abnormal phenotypes	References
*SHANK3*	Core postsynaptic scaffold protein stabilizing synaptic structure	Loss of function disrupts synaptic signal transmission	Phelan-McDermid syndrome, sporadic ASD	[81, 82]
*NLGN3*	Postsynaptic adhesion molecule balancing neural circuit excitability	Mutations impair synaptic plasticity and circuit stability	Social deficit, stereotyped behavior, cognitive impairment	[87, 113]
*CNTNAP2*	Synaptic adhesion protein modulating embryonic neurodevelopment	Variations hinder neural circuit formation and damage social-related brain regions	ASD, epilepsy, intellectual disability	[88, 114, 115]
*NRXN1*	Presynaptic adhesion molecule coordinating synaptic maturation	Heterozygous deletion leads to synaptic developmental defects	Language delay, epilepsy, neurodevelopmental anomalies	[89]
*MECP2*	Methyl-binding transcriptional regulator sustaining neuronal homeostasis	Dysfunction causes dendritic malformation and neurodevelopmental defects	Motor abnormality, neurodevelopmental disorder, ASD-like behaviors	[92]
*KDM5*	Histone demethylase regulating neuroimmune homeostasis	Downregulation activates central inflammatory pathways and induces immune disturbance	Abnormal social behaviors, chronic neuroinflammation	[95]
*FMR1*	Encodes FMRP and maintains homeostasis of synaptic-related mRNA	FMRP deficiency activates mGluR5 pathway and triggers abnormal synaptic plasticity	Social impairment, stereotyped behavior, synaptic dysfunction	[101]
*UBE3A*	E3 ubiquitin ligase regulating synaptic remodeling in a dose-dependent manner	Bidirectional expression imbalance disrupts synaptic pruning	Angelman syndrome, ASD, synaptic homeostatic disorder	[106–108]
*ANK3*	Neuronal cytoskeleton protein ensuring stable nerve impulse conduction	Functional loss induces excitatory-inhibitory imbalance	Impaired learning and memory, dyskinesia	[111, 112]

*ASD* Autism Spectrum Disorder, *FMRP* Fragile X Mental Retardation Protein, *mGluR5* metabotropic glutamate receptor 5, *E3* E3 ubiquitin ligase

### Characteristics of major genetic patterns

Polygenic inheritance is the most common model. ASD is not due to a single gene but to the cumulative effect of multiple risk genes; when the genetic load crosses a threshold, disease develops [116]. This model explains both the strong family clustering of ASD and why most sporadic cases have no clear family history. Studies on PRS show that higher PRS values are linked to higher ASD risk and more severe clinical symptoms, directly supporting the additive effect of common variants. In addition, gene–gene interactions (whether synergistic or antagonistic) further influence disease risk [65].

Yet a high polygenic load does not guarantee disease. Incomplete penetrance means that among individuals carrying the same variant, only some show typical ASD symptoms; others have no obvious signs or only mild traits [117]. This reflects the influence of epigenetic modifications, environmental triggers, and modifier genes. It also explains why symptom severity can vary within the same family and confirms that ASD arises from both genetic factors and environmental influences [118].

Beyond penetrance differences, ASD shows significant genetic heterogeneity among patients. Even with similar clinical features, individuals may carry completely different variants, rare point mutations, CNVs, common SNPs, or non-coding changes, acting alone or in combination [119]. This heterogeneity is a major barrier to establishing a unified genetic diagnostic standard and to developing targeted therapies. Current research on subtyping focuses largely on disentangling this complexity.

Notably, beyond these complex patterns, only about 5% to 10% of ASD cases follow classical Mendelian inheritance patterns, such as autosomal dominant, autosomal recessive, or X-linked recessive. These cases are mostly due to rare, highly penetrant single-gene variants [120]. For example, mutations in *NLGN3* and *NLGN4* follow X-linked inheritance, which helps explain the characteristic 4:1 male-to-female ratio in ASD [121]. Dominant mutations in *ARID1B* are often associated with severe intellectual disability and are a typical autosomal dominant form of ASD [122].

### Gene-environment interaction

Genetic susceptibility is only part of the ASD story. Gene-environment interactions (G × E) complete the picture and have become a major focus of recent research. Genetic background sets the baseline risk for ASD, but adverse early-life exposures, especially during the critical prenatal window, can lower the disease threshold, activate abnormal neurodevelopmental pathways, and ultimately trigger or worsen symptoms [123, 124]. These interactions are largely mediated by epigenetic changes. Environmental stimuli alter DNA methylation, histone modifications, and non-coding RNA expression, thereby regulating the transcription of neurodevelopmental risk genes without changing DNA sequences and impairing neuronal proliferation, differentiation, migration, and synaptic remodeling [125]. During this period, maternal exposures can cross the placental barrier and directly affect the fetal brain. Fetuses carrying genetic susceptibility variants are far more vulnerable to such environmental insults [126, 127].

Such combined effects are not simply additive. Instead, they show high specificity, dose dependence, and synergistic amplification. Different environmental risk factors target specific susceptibility genes and act through distinct pathological pathways. For example, maternal infection during pregnancy can activate the TLR4 inflammatory pathway and impair synaptic plasticity [128]. Furthermore, insufficient vitamin D intake and VDR gene variants together suppress normal neuronal differentiation and raise disease risk in offspring [129]. Moreover, the duration and intensity of environmental exposure can interact synergistically with genetic load. When both risk factors are present, ASD risk rises sharply compared with either factor alone [130, 131]. G × E interactions also shape the phenotypic heterogeneity of ASD. Even with similar genetic backgrounds, patients can differ greatly in symptom severity and comorbidity rates due to varying environmental exposures [132].

Although the importance of G × E is widely acknowledged, current research has notable limitations. Most studies are retrospective, so establishing definitive causality remains difficult. Measuring environmental exposures precisely remains challenging, and the high genetic heterogeneity of ASD hinders the development of a universal G × E model [133]. Moving forward, large prospective birth cohorts combined with multi-omics techniques will help identify high-risk gene-environment pairs and clarify the underlying molecular mechanisms. These advances may offer new opportunities for preconception and early prenatal screening and intervention.

Overall, ASD genetic research has evolved from basic association analyses to in-depth mechanistic exploration. Many risk genes, inheritance patterns, and gene-environment mechanisms have been identified. Nevertheless, incomplete penetrance, profound heterogeneity, and uncharacterized regulatory pathways still await further investigation. Addressing these gaps will be a core direction for future research and clinical translation.

## Molecular mechanism of ASD

Recent years have witnessed substantial breakthroughs in elucidating the molecular mechanisms of ASD, enabled by advances in genomics, single-cell sequencing, and brain organoid technologies. These studies have established that ASD is characterized by widespread pathological abnormalities across multiple levels, including synaptic function, signaling pathway regulation, neurodevelopmental processes, neuroimmune homeostasis, and neural circuit balance. This chapter systematically reviews these core research advances, integrating multidimensional experimental and clinical evidence to deeply elucidate the pathophysiological basis of autism. It further aims to provide a robust theoretical foundation for the development of precision diagnostic biomarkers and therapeutic targets, and the design of targeted intervention strategies.

### Synaptic structure and function

Synapse dysfunction is the most central and best understood molecular mechanism in ASD. Both patient brain tissue and animal models show abnormal synapse morphology, altered synapse number, and impaired signal transmission. The severity of these changes correlates closely with core symptoms such as social deficits and repetitive behaviors [134, 135].

This dysfunction starts with abnormal synapse structure. Synaptic adhesion molecules govern the formation and stabilization of connections between neurons, and their dysfunction directly impedes normal synaptic development [136]. At the level of synaptic adhesion, variants in *Nectin-1*, *Neuroligin*, and *Neurexin* family molecules are frequently detected in individuals with autism. These variants compromise synaptic connection stability and reduce the number of functional synapses. Among them, Neuroligin and Neurexin proteins are typical contributors to synaptic pathology when mutated [137]. More specifically, mutations in *NLGN3* and *NLGN4*, whether loss-of-function or gain-of-function, impair synaptic adhesion and decrease functional synapse numbers [85]. *NRXN1* deletions impair signaling between pre- and postsynaptic membranes, alter neurotransmitter release kinetics, and impede normal neural signal transmission [138]. Adhesion molecule variants such as *CNTNAP2* are also found at high rates in autism populations. These variants not only weaken synaptic adhesion and connection stability, but also interact with environmental factors like prenatal tobacco exposure, further exacerbating synapic dysfunction and aggravating social and communication deficits [139].

Unstable structure then impairs postsynaptic signal integration. SHANK family scaffold proteins are core components of the postsynaptic density (PSD). They bring together glutamate receptors, signaling molecules, and cytoskeletal proteins, and they help shape synapse structure and regulate synaptic function. Mutations in these genes are a major genetic risk factor for autism [140]. SHANK3 mutations in particular cause the PSD structure to collapse. This disrupts the proper placement of AMPA and other glutamate receptors on the postsynaptic membrane. Synaptic signaling becomes markedly impaired, and excitatory-inhibitory balance in neural circuits is disrupted [141]. Abnormal SHANK proteins also impair synaptic plasticity processes such as long-term potentiation and long-term depression, which compromises the brain's ability to integrate and transmit neural signals [142].

This signal integration failure ultimately disrupts the balance of neurotransmitter release and clearance. *Syntaxin 1A* abnormalities slow neurotransmitter release [143]. Defects in glutamate transporters cause glutamate to accumulate in the synaptic cleft, triggering excitotoxicity [144]. GABA transporter dysfunction alters inhibitory neurotransmitter metabolism, exacerbating the excitatory-inhibitory imbalance in neural circuits [7].

These molecular abnormalities do not act alone. They form a complex regulatory network that together disrupts synapse development and signal transmission, which helps explain why ASD symptoms are so varied. Different clinical subtypes show distinct patterns of synaptic dysfunction, offering potential directions for precison subtyping. Future work needs to bridge basic and clinical research, using animal models to test interventions, identify subtype-specific markers, and move synapse-targeted strategies toward clinical use.

### Key signaling pathways

During brain development, several classic signaling pathways coordinately regulate neuronal proliferation, differentiation, migration, and synaptogenesis. Dysregulation of these pathways, whether hyperactivation or hypoactivation, is a key mechanism underlying the neurodevelopmental abnormalities observed in autism. To date, four pathways have been most strongly implicated in autism: mTOR, Ras-ERK-MAPK, Wnt/β-catenin, and Notch. Each can function independently to modulate neurodevelopment and synapse function, and they can also interact with each other to contribute to pathogenesis.

The mTOR pathway is the central regulator of cell growth and synaptic plasticity. When this pathway is hyperactivated, it causes synaptic dysfunction and abnormal brain development. In people with autism, negative regulators such as *TSC1*, *TSC2*, and *PTEN* are often mutated. These mutations relieve inhibition of the pathway, causing neuronal hyperproliferation and dendritic overgrowth. At the same time, they impair autophagy, leading to accumulation of synaptic proteins [145, 146]. The mTOR pathway also controls glutamate receptor trafficking and membrane insertion. When abnormally active, it strengthens long-term potentiation and weakens long-term depression, disrupting circuit homeostasis [147, 148]. In addition, environmental factors such as poor nutrition or toxin exposure during pregnancy can modulate mTOR activity and interact with genetic risk to worsen symptoms [149, 150].

Unlike mTOR, the Ras-ERK-MAPK pathway focuses more on neuronal proliferation and synaptogenesis. Mutations in genes such as *NF1*, *KRAS*, and *BRAF* can hyperactivate this pathway, disrupting the normal kinetics of cell proliferation in neural precursors and leading to abnormalities in the density and laminar organization of cortical neurons [151]. This pathway also regulates synapse adhesion molecules and scaffold proteins. When dysregulated, it impairs normal synapse formation and stability, leading to impaired synaptic transmission [152]. Notably, this pathway converges with mTOR. When both are abnormally active, neurodevelopmental abnormalities are exacerbated [152, 153].

The Wnt/β-catenin pathway plays an essential role during early brain development. It precisely regulates neural stem cell self-renewal, neuronal migration, and synaptogenesis. Whether hyperactivated or hypoactivated, this pathway causes defects in brain development [154, 155]. Abnormal expression of ligands such as *WNT2* and *WNT7A* [156], or mutations in negative regulators such as *APC* and *GSK-3β*, can impair normal development of core brain regions including the cortex and cerebellum, causing neurons to fail to complete migration and synaptic density to decrease markedly [157]. Abnormal Wnt signaling also interferes with the development of GABA neurons, disrupting the balance between excitation and inhibition in neural circuits. This is closely associated with social deficits and repetitive behaviors [158].

The Notch pathway primarily determines neural stem cell fate and neuronal lineage specification. When dysregulated, it disrupts neuronal subtype specification, which compromises overall circuit function [159]. Variants in genes encoding Notch receptors and their ligands, such as Delta-like and Jagged, cause abnormal fluctuations in pathway activity. On one hand, this disrupts the equilibrium between excitatory and inhibitory neuron production. On the other hand, it impairs normal synapse development and plasticity [10]. Furthermore, the Notch pathway mediates epigenetic changes, indirectly modulating the expression of genes required for brain development and thus contributing to autism through multiple mechanisms [160].

**Fig. 3:**
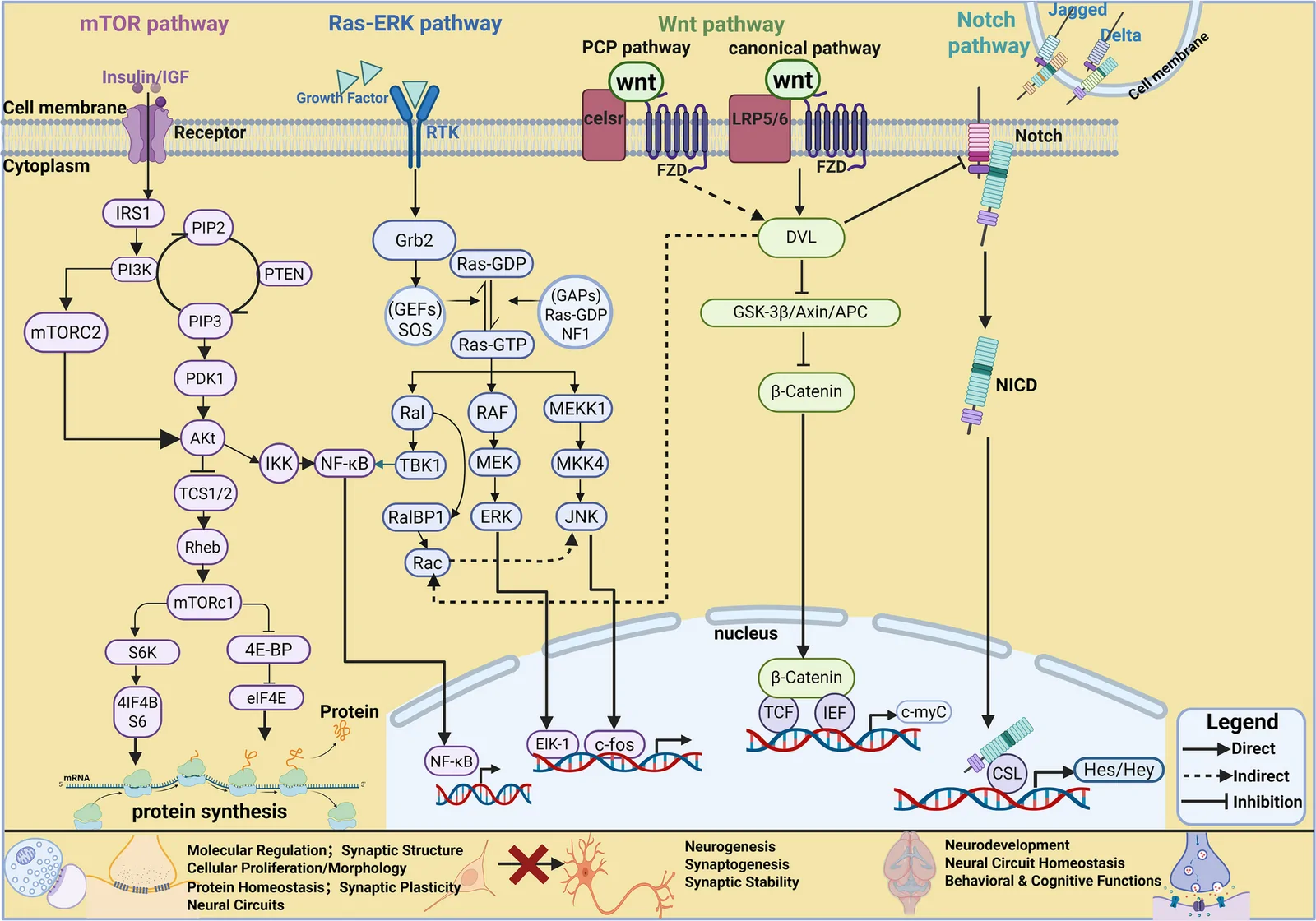
Schematic illustration of four core signaling pathways implicated in ASD pathogenesis and their inter-pathway crosstalk. (1) The PI3K-Akt-mTOR pathway. Insulin/IGF ligands bind to their receptors, recruiting IRS1 and activating PI3K, which phosphorylates PIP2 to generate PIP3. PIP3 recruits PDK1 and Akt to the membrane; PDK1 phosphorylates Akt, and mTORC2 phosphorylates Akt. Activated Akt phosphorylates IKK, leading to NFκB-mediated transcription. Akt also inhibits the TSC1/TSC2 complex, thereby relieving its suppression on Rheb and activating mTORC1. mTORC1 promotes protein synthesis through phosphorylation of S6K and 4E-BP1, the latter releasing eIF4E from translational inhibition. (2) The Ras-ERK-MAPK pathway. Growth factors activate RTKs, recruiting Grb2 and GEFs to catalyze Ras-GDP to Ras-GTP exchange. Ras-GTP activates multiple downstream branches: the Ral-TBK1 axis promoting NFκB transcription; the RAF-MEK-ERK cascade phosphorylating transcription factors including ELK1 and c-fos; and the MEKK1-MKK4-JNK pathway, wherein MEKK1 phosphorylates and activates MKK4, which in turn phosphorylates JNK, leading to JNK nuclear translocation and phosphorylation of transcription factors to alter gene expression. Additionally, RalBP1-mediated Rac activation indirectly promotes JNK. Ras-GTP also cross-activates PI3K, linking the Ras-ERK and mTOR pathways. (3) The Wnt signaling pathway. Left: non-canonical Wnt/PCP pathway—Wnt ligands bind FZD receptors and Celsr co-receptors, activating DVL, which indirectly affects Rac. Right: canonical Wnt/β-catenin pathway—Wnt ligands bind FZD receptors and LRP5/6 co-receptors, activating DVL, which inhibits the GSK-3β/Axin/APC destruction complex, stabilizing β-catenin for nuclear translocation and TCF/LEF-mediated transcription of target genes including c-myc. (4) The Notch signaling pathway. Delta/Jagged ligands bind Notch receptors, triggering sequential cleavage to release NICD, which translocates to the nucleus and binds the transcription factor CSL to activate transcription of Hes/Hey target genes. Crosstalk nodes: DVL indirectly affects Rac, linking the Wnt/PCP and Ras-ERK pathways; DVL inhibits Notch signaling, linking the Wnt and Notch pathways; Ras-GTP activates PI3K and promotes NFκB via the Ral-TBK1 axis, linking the Ras-ERK and mTOR pathways. Collectively, these pathway convergences alter gene transcription and protein synthesis, disrupt synaptic development and plasticity, induce excitation-inhibition imbalance, impair neuronal function, and constitute the core pathological hub of ASD. Solid arrows indicate direct activation; dashed arrows indicate indirect regulation; blunt-ended lines indicate inhibition. Graphics created with BioRender. ASD Autism Spectrum Disorder, PI3K Phosphoinositide 3-Kinase, Akt Ak strain transforming/Protein Kinase B, IGF Insulin-like Growth Factor, IRS1 Insulin Receptor Substrate 1, PIP2 Phosphatidylinositol 4,5-Bisphosphate, PIP3 Phosphatidylinositol [3–5]-Trisphosphate, PDK1 3-Phosphoinositide-Dependent Protein Kinase 1, IKK IκB Kinase (Inhibitor of Nuclear Factor kappa B Kinase), NFκB Nuclear Factor Kappa B, TSC1/TSC2 Tuberous Sclerosis Complex 1/2, Rheb Ras Homolog Enriched in Brain, mTORC1 mechanistic Target of Rapamycin Complex 1, mTORC2 mechanistic Target of Rapamycin Complex 2, S6K Ribosomal Protein S6 Kinase, 4E-BP1 Eukaryotic translation Initiation Factor 4E-Binding Protein 1, eIF4E Eukaryotic translation Initiation Factor 4E, ERK Extracellular signal-Regulated Kinase, MAPK Mitogen-Activated Protein Kinase, RTKs Receptor Tyrosine Kinases, Grb2 Growth Factor Receptor-Bound protein 2, GEFs Guanine nucleotide Exchange Factors, GDP Guanosine Diphosphate, GTP Guanosine Triphosphate, Ras Rat Sarcoma viral oncogene homolog, Ral Ras-like protein, TBK1 TANK-Binding Kinase 1, RAF Rapidly Accelerated Fibrosarcoma, MEK Mitogen-activated protein Kinase Kinase (MAPK/ERK Kinase), ELK1 ETS Like-1, MEKK1 MEK Kinase 1 (MAP Kinase Kinase Kinase 1), MKK4 MAP Kinase Kinase 4 (Mitogen-activated protein Kinase Kinase 4), JNK c-Jun N-terminal Kinase, RalBP1 Ral-Binding Protein 1, Rac Ras-related C3 botulinum toxin substrate, Wnt Wingless-related integration site, PCP Planar Cell Polarity, FZD Frizzled, Celsr Cadherin EGF LAG Seven-Pass G-Type Receptor, DVL Dishevelled, LRP5/6 Low-Density Lipoprotein Receptor-related Protein 5/6, GSK-3β Glycogen Synthase Kinase-3 beta, APC Adenomatous Polyposis Coli, TCF/LEF T Cell Factor/Lymphoid Enhancer Factor, NICD Notch Intracellular Domain, CSL CBF1/Suppressor of Hairless/Lag-1 (C-promoter Binding Factor 1), Hes/Hey Hairy and Enhancer of Split/Hairy/Enhancer-of-split related with YRPW motif

These four pathways have distinct functions but also interact to maintain normal neurodevelopment. mTOR dysregulation is characterized by synaptic protein accumulation and plasticity imbalance. Ras-ERK-MAPK dysregulation is typically triggered by genetic mutations and tends to converge with other pathways. Wnt/β-catenin dysregulation primarily impairs brain region development and inhibitory neuron function. Notch dysregulation primarily disrupts the ratio of neuronal subtypes. All of these pathways contribute to neurodevelopmental abnormalities by disrupting neural development, compromising synaptic architecture, or mediating epigenetic modifications. When pathway crosstalk and environmental factors are considered, the clinical heterogeneity is further exacerbated. The physiological roles and pathogenic mechanisms of these core ASD-related signaling pathways are summarized in Table 2. Understanding exactly how these pathways are dysregulated and interact will facilitate the identification of diagnostic biomarkers and guide the development of targeted therapeutics (Fig. 3).

**Table 2:** Core signaling pathways implicated in ASD: physiological functions, pathogenic mechanisms and key references

Signaling Pathway	Core Physiological Function	Abnormal Mechanisms Linked to ASD	References
mTOR signaling pathway	Modulates cell growth, metabolism and synaptic plasticity, involving neuronal proliferation, dendritic branching, autophagy, glutamate receptor synthesis and trafficking	1. Mutations of TSC1, TSC2 and PTEN drive overactivation of the pathway 2. Excessive neuronal proliferation and aberrant dendrite growth occur; impaired autophagy accumulates synaptic proteins and damages synaptic structure and function 3. Altered glutamate receptors enhance LTP and attenuate LTD, disrupting neural circuits 4. Prenatal adverse factors and genetic variants jointly disturb pathway activity and exacerbate pathological manifestations	[8, 146, 149, 150]
Ras-ERK-MAPK signaling pathway	Modulates neuronal proliferation, differentiation and synaptogenesis, and regulates neural progenitor cells as well as the expression of synaptic adhesion molecules and scaffold proteins	1. Mutations of NF1, KRAS and BRAF genes lead to aberrant pathway activation 2. Disrupt proliferation and differentiation of neural progenitor cells, causing abnormal neuronal density and disordered lamination in the cerebral cortex 3. Abnormal expression of synaptic adhesion molecules and scaffold proteins impairs synaptic formation and stability, and hinders neural signal transmission 4. Synergistically interacts with the mTOR pathway to exacerbate pathological damage	[151–153, 161]
Wnt/β-catenin signaling pathway	A core pathway for embryonic brain development, controlling neural stem cell self-renewal, neuronal migration and synaptogenesis	1. Dysregulated WNT2/WNT7A or mutated APC/GSK-3β induces aberrant pathway activity 2. Disrupt cortical and cerebellar development, impairing neuronal migration and synaptogenesis 3. Defective GABAergic function disturbs excitation-inhibition balance, contributing to social deficits and repetitive behaviors	[154, 155, 157, 158]
Notch signaling pathway	Governs neural stem cell differentiation and neuronal fate determination, participates in synaptogenesis and synaptic plasticity, and mediates epigenetic modification	1. Variations in Delta-like and Jagged ligands/receptors induce aberrant Notch pathway activity 2. Disrupt neural stem cell differentiation, altering the ratio of glutamatergic and GABAergic neuronal subtypes 3. Impairs synaptic formation and plasticity, disrupting neural signal transmission 4. Alter epigenetic modification and neurodevelopmental gene expression, promoting ASD pathogenesis	[10, 159, 160]

*mTOR* mechanistic Target of Rapamycin, *ERK* Extracellular signal-Regulated Kinase, *MAPK* Mitogen-Activated Protein Kinase, *TSC1/TSC2* Tuberous Sclerosis Complex 1/2, *PTEN* Phosphatase and Tensin Homolog, *LTP* Long-Term Potentiation, *LTD* Long-Term Depression, *NF1* Neurofibromatosis type 1, *KRAS* Kirsten Rat Sarcoma viral oncogene homolog, *BRAF* B-Raf (v-Raf murine sarcoma viral oncogene homolog B1), *Wnt* Wingless-related integration site, *APC* Adenomatous Polyposis Coli, *GSK-3β* Glycogen Synthase Kinase-3 beta

### Neurodevelopmental process

ASD is a neurodevelopmental disorder that begins in the embryo and continues through early childhood. The core problems are dysregulated neuronal proliferation and differentiation, failed migration, aberrant synaptogenesis, and disrupted neural circuit formation. Both genetic risk and environmental factors shape this process, leading to lasting damage in brain structure and function. New tools such as brain organoids and single-cell sequencing have helped elucidate how this damage unfolds [162].

The first problem appears early in the embryo, when neural stem cells fail to proliferate and differentiate properly. Mutations or abnormal expression of genes such as *SOX2, PAX6*, and *SHH* disrupt the equilibrium of cell division, altering neuronal density in key regions including the cortex, cerebellum, and hippocampus [163, 164]. Abnormal *SOX2* impairs stem cell renewal, causing abnormal neuronal density across the brain [165]. *SHH* defects reduce the number of Purkinje cells in the cerebellum, which is associated with poor motor coordination and social cognition in patients [166]; *PAX6* mutations disrupt cortical neuronal differentiation, disrupting the equilibrium between excitatory and inhibitory cells and predisposing to later circuit problems [167, 168].

This dysregulated proliferation and differentiation then leads to failed neuron migration. For proper cortical lamination and functional circuit formation, neurons must migrate in an orderly manner. When mutations affect genes such as *DCX, LIS1,* and *RELN*, migration stalls, producing a disorganized cortex and ectopic neurons [169]. *RELN* encodes the Reelin protein that guides migration speed and direction; when mutated, neurons stop moving and settle incorrectly, disrupting intracortical connectivity [170]. *DCX* defects impair migration, producing abnormal neuron distribution in the hippocampus and amygdala, regions central to emotion and social cognition, and directly impairing the development of these functions [171].

After migration fails, synapse formation and pruning become the main source of symptoms. Normally, the infant brain rapidly generates numerous synapses, then depends on microglia-mediated pruning of redundant or dysfunctional synapses, thereby establishing mature, stable circuits. In ASD, this process is disrupted: synaptogenesis is impaired, and pruning is either excessive or insufficient, resulting in aberrant synaptic density [172]. Mutations in risk genes such as *SHANK3, NLGN4*, and *MECP2* downregulate synaptogenic molecules and also compromise microglial function, disrupting pruning and impeding circuit maturation. These alterations manifest as delayed social communication and cognitive deficits [92, 141, 173].

Beyond these steps, delayed myelin development also contributes. Myelin wraps nerve fibers to accelerate signal transmission and enable efficient coordination between brain regions. When myelin development is impaired, signals slow down and overall information processing declines [174]. Abnormal expression of myelin genes such as *MBP* and *PLP1* leads to insufficient myelin and delayed myelination, especially in the cortex and corpus callosum. This is closely associated with low cognitive ability and impaired information integration in patients [175].

ASD-related neurodevelopmental problems exhibit distinct temporal and regional specificity, accounting for the marked clinical heterogeneity. Overall, pathological alterations unfold continuously from the embryonic period through early childhood, with dysregulated proliferation and differentiation, failed migration, aberrant synaptogenesis and pruning, and delayed myelination as core pathological features. These problems are interconnected and sequentially compounded: early defects in primitive nerve cells predispose to later synapse and myelin problems, while later structural disorganization ultimately disrupts circuit function and manifests as core clinical symptoms.

### Neuroimmunology and inflammatory response

In recent years, the role of neuroimmune dysfunction and chronic brain inflammation in autism has gained increasing attention. Research has shown that ongoing low-level inflammation in the central nervous system, together with imbalanced immune function throughout the body, acts as a key contributing factor. These immune abnormalities can interact with genetic susceptibility and environmental risks, disrupting normal brain development and synapse function, and have opened up new directions for immune-based interventions [176, 177].

Chronic brain inflammation is a core immune feature of autism. It mainly manifests as microglial hyperactivation, astrogliosis, and elevated inflammatory mediators. Microglia are the brain's resident immune cells and also mediate synaptic pruning. In people with autism and in animal models, microglia remain chronically activated, releasing inflammatory mediators such as IL-6, IL-1β, and TNF. These signals can directly compromise neuronal viability, impair synaptogenesis and plasticity, and disrupt neurotransmitter synthesis and release, ultimately disrupting the equilibrium of neural circuits [178, 179]. Abnormal astrocyte growth also leads to gliotic scarring, destabilizing the microenvironment and impairing metabolic support to neurons, which exacerbates neurodevelopmental pathology [180].

Brain inflammation does not exist in isolation. It is closely associated with immune imbalance in the rest of the body, and the two perpetuate a self-reinforcing cycle. Individuals with ASD frequently exhibit altered T/B cell subset ratios in their blood, along with elevated inflammatory markers. These peripheral immune signals can cross the blood–brain barrier and activate glial cells in the brain, amplifying the inflammatory response [181, 182]. Against this backdrop, maternal immune activation has emerged as the most studied pregnancy-related risk factor. When a mother experiences infection or allergic stress during pregnancy, her immune system mounts an exaggerated response, and inflammatory signals undergo transplacental transfer, directly interfering with fetal brain development and increasing offspring susceptibility to autism [183]. Population data show that severe maternal viral infection during pregnancy can increase this risk by roughly 30% [184]. Animal studies confirm that maternal immune activation causes fetal microglia to prematurely activate, disrupts neuronal migration, and impairs synaptogenesis, leading to autism-like behaviors in offspring. This damage interacts with susceptibility genes such as *TLR4* to exacerbate neurodevelopmental pathology [128, 185].

Beyond inflammation, autoimmunity represents a separate disease pathway. Individuals with ASD exhibit significantly elevated prevalence of brain-directed autoantibodies compared with healthy individuals. These antibodies can bind directly to functional proteins on the neuronal surface, including synaptic proteins and neurotransmitter receptors, compromising neuronal integrity, attenuating synaptic transmission, and disrupting circuit function [186, 187]. Anti-NMDAR antibodies, for example, bind to glutamate receptors on the postsynaptic membrane and impair excitatory neurotransmission, which is directly associated with cognitive impairment in patients [188]. Gut microbiome imbalance provides an additional mechanism, mediated by peripheral factors. Disrupted gut bacteria can modulate systemic immune homeostasis through the gut-brain axis and indirectly trigger brain inflammation. Based on this, restoring gut microbiome balance and improving immune function has become a promising adjunctive therapeutic strategy [189].

### Excitation-inhibition balance

Normal brain function depends on a dynamic balance between excitatory and inhibitory neurons. When this balance is disrupted, it constitutes a common pathological hallmark that links synaptic dysfunction, abnormal signaling pathways, and neurodevelopmental defects. It is also a cardinal end-stage pathological feature in autism [190, 191]. In individuals with ASD, this imbalance is frequently observed, with a predominant shift toward enhanced excitatory signaling and attenuated inhibitory function [192]. This manifests as hyperexcitable circuits and disrupted synchronous firing, which ultimately leads to core symptoms such as impaired social communication and repetitive behaviors. The underlying mechanism primarily involves dysfunction of the glutamatergic and GABAergic systems [193].

The imbalance starts with overactivity of the glutamate system. Glutamate is the main excitatory neurotransmitter in the central nervous system. Dysregulation of its synthesis, release, transport, or receptor function collectively results in excessive excitatory signaling [194]. In individuals with ASD, altered expression of enzymes involved in glutamate metabolism frequently results in substantial accumulation in the synaptic cleft. Mutations in receptor genes such as *GRIN2A* and *GRIN2B* increase receptor activity, overstimulating excitatory transmission and inducing neuronal hyperexcitability [195]. In addition, abnormalities in synaptic scaffolding proteins such as *SHANK3* disrupt the normal positioning of glutamate receptors on the membrane, further amplifying excitatory signals and disrupting the equilibrium [141].

At the same time, GABAergic dysfunction is the primary mechanism for attenuated inhibition. GABA is the main inhibitory neurotransmitter. During neurodevelopment in ASD, GABAergic neurons often show impaired development, reduced numbers, and abnormal migration, directly limiting the availability of inhibitory signals. Expression of key synthetic enzymes such as GAD65 and GAD67 is reduced, markedly lowering GABA production. Mutations in receptor genes including *GABRB3* and *GABRG2* weaken inhibitory signaling. Meanwhile, abnormal GABA transporters accelerate clearance of the inhibitory transmitter from the synaptic cleft. Together, these pathological changes progressively impair inhibitory capacity, rendering it unable to suppress overactive excitatory signals [134, 195].

This imbalance does not exist in isolation. It is shaped by multiple upstream mechanisms. Overactivation of the mTOR pathway can both increase glutamate receptor expression and impair normal development of inhibitory neurons, exacerbating the imbalance bidirectionally [147, 196]. During central inflammation, activated glial cells release pro-inflammatory factors that suppress GABAergic neuron function while strengthening glutamatergic signaling, creating a vicious cycle driven by inflammation [197, 198]. Because genetic background, environmental exposures, and clinical symptoms vary across individuals, this imbalance exhibits marked interindividual heterogeneity. Some show mainly excitatory hyperactivity, others mainly inhibitory weakness, and still others a combination of both.

From a clinical standpoint, this imbalance offers a valuable therapeutic target. Drugs that modulate glutamatergic and GABAergic systems to restore circuit balance have demonstrated promising efficacy in animal models. Administration of GABA receptor agonists or inhibition of overactive glutamate receptors both significantly improve social deficits and repetitive behaviors in animal models [199, 200]. In addition, adjusting the excitation-inhibition balance through epigenetic modifications and approaches that modulate neuroplasticity offers novel avenues for personalized treatments [201].

Overall, the molecular pathogenesis of autism forms a complex pathological network that spans multiple structures, pathways, and systems. Synaptic structural and functional abnormalities are the most direct core features. Imbalanced activity across various signaling pathways serves as a key upstream regulatory mechanism. Neurodevelopmental defects from the embryonic stage to infancy are the congenital root of the disorder. Neuroimmune dysregulation and chronic inflammation act as important acquired contributing factors. Excitation-inhibition imbalance integrates all of these pathological insults and serves as the common final outcome where various abnormalities converge. The underlying mechanisms, pathological manifestations, and clinical relevance of excitation-inhibition (E/I) balance disturbance in ASD are summarized in Table 3. These mechanisms interact and overlap, combined with genetic and environmental influences, ultimately producing the highly heterogeneous clinical profile of autism. Future research using multi-omics approaches and organoid disease models will further clarify how these mechanisms connect and identify core regulatory targets. This will establish a solid molecular foundation for early screening, precise diagnosis, and individualized targeted therapy.

**Table 3:** Mechanisms, pathological manifestations and clinical relevance of excitation-inhibition (E/I) balance disturbance in ASD

Core module	Key mechanism	Specific molecular/pathway/cellular abnormality	Pathological manifestation and impact on E/I balance	References
Core status of E/I balance in ASD	E/I balance maintains normal neural circuit function. Its disruption acts as the core hub linking multi-dimensional pathological abnormalities	Homeostatic imbalance of excitatory and inhibitory neuronal circuits	Aberrant circuit excitation and disordered signal synchronization trigger core autistic clinical symptoms	[190–193]
Glutamatergic system abnormality	Abnormal neurotransmitter metabolism, receptor function and synaptic structure cause excessive excitatory signaling	Dysregulated glutamine synthetase expression; NMDAR and AMPAR gene mutations; SHANK3 abnormality induced receptor mislocalization	Glutamate accumulation in synaptic clefts elevates neuronal excitability and shifts E/I balance toward excitation	[141, 194, 195]
GABAergic system abnormality	Defects in neuronal development, neurotransmitter synthesis, receptors and transporters weaken inhibitory function	Impaired inhibitory neuronal development; downregulated GAD65/GAD67; dysfunctional inhibitory receptors and transporters	Insufficient GABA synthesis and blocked signal transmission greatly reduce central inhibitory regulation	[134, 194, 195]
Disturbed upstream regulation of E/I balance	Signaling pathways and neuroinflammation jointly disrupt balance homeostasis	Excessive mTOR pathway activation; pro-inflammatory factors released by activated microglia	Pathway imbalance and neuroinflammation mutually aggravate E/I disturbance and form a persistent pathological cycle	[147, 196, 197]
Heterogeneity of E/I imbalance	Genetic background, environmental exposure and clinical phenotypes lead to individual pathological differences	Combinations of genetic susceptibility loci and acquired environmental triggers	Form pathological subtypes including isolated hyper-excitation, isolated hypo-inhibition and combined dual abnormalities	[202]
Clinical intervention value of E/I balance	Targeted regulation of two major neurotransmitter systems restores circuit homeostasis	GABA receptor agonists, glutamate receptor inhibitors and epigenetic modulation strategies	Effectively ameliorates autistic-like behaviors in animal models and provides viable directions for targeted clinical therapy	[199–201]

*ASD* Autism Spectrum Disorder, *E/I* Excitation/Inhibition, *NMDAR* N-Methyl-D-Aspartate Receptor, *AMPAR* α-Amino-3-hydroxy-5-Methyl-4-isoxazolepropionic Acid Receptor, *SHANK3* SH3 and Multiple Ankyrin Repeat Domains 3, *GABA* Gamma-Aminobutyric Acid, *GAD65/GAD67* Glutamate Decarboxylase 65/67, *mTOR* mechanistic Target of Rapamycin

## Clinical characteristics and animal model application of autism

This chapter covers two main topics: the clinical features of ASD and the role of animal models in studying it. ASD is a neurodevelopmental disorder that exhibits marked interindividual heterogeneity, with symptoms that evolve across the lifespan. Its core features are persistent difficulties in social communication and interaction, alongside repetitive behaviors and narrow interests. Many individuals also have co-occurring neuropsychiatric conditions, which expand the clinical spectrum and contribute to functional heterogeneity. Understanding these core symptoms, subtyping distinctions, common comorbidities, and longitudinal disease progression is essential. This facilitates clinical recognition and guides both diagnosis and treatment.

Because ASD involves complex human social and cognitive functions, researchers cannot perform invasive mechanistic studies or test new therapies directly in patients. Animal models fill this gap. They allow scientists to study basic biology, explore disease mechanisms, and screen potential drugs, while replicating the neurodevelopmental changes, behavioral abnormalities, and molecular abnormalities seen in individuals with ASD. This chapter first reviews the diverse clinical features of ASD. It then describes how current animal models are built, what behavioral and biological traits they show, what strengths they offer, and when they are most useful. By connecting clinical observations with experimental findings, this chapter shows why animal models are central to understanding ASD and moving research toward better diagnosis and personalized treatment [203, 204].

### Core symptoms

DSM-5 divides the core symptoms of ASD into two domains: persistent deficits in social communication and interaction, and restricted, repetitive patterns of behavior, interests, or activities. Both begin early in development and continue to impair social, academic, and daily functioning. They form the basis of clinical diagnosis. Between the two, social communication deficits are the most distinctive feature of ASD. They span verbal expression, nonverbal exchange, and reciprocal social engagement. They also serve as the key marker that distinguishes ASD from other neurodevelopmental conditions.

These social difficulties unfold across several levels. At the most immediate level, individuals with ASD exhibit deficits in social-emotional reciprocity. They demonstrate impaired engagement in reciprocal social interaction, are less responsive to others' verbal or physical cues, and rarely initiate sharing of interests, feelings, or everyday experiences. In infancy and early childhood, this often manifests as limited eye contact, failure to respond to their name, and a lack of spontaneous social smiling. As children enter school age, the phenotype evolves to include avoiding peer interaction, refusing to join cooperative games, and showing a clear preference for solitary play [50, 205, 206].

Nonverbal communication is also markedly impaired. Many individuals fail to read or use eye contact, facial expressions, and gestures in socially expected ways. Their eye contact may be unusual or absent, facial expressions remain limited, and hand gestures are either missing or repetitive. Furthermore, many individuals also show atypical sensory processing. They may be overly sensitive or under-sensitive to light, sound, or touch. This further impairs nonverbal communication and compromises social engagement [4].

Beyond these surface behaviors, individuals with ASD also exhibit deficits in the higher-order cognitive process of establishing and maintaining relationships. They often show impaired theory of mind: they have difficulty comprehending social norms, reading others' intentions, and inferring mental states. They show limited empathy and have difficulty modulating their behavior to adapt to dynamic social contexts. As a result, establishing and maintaining meaningful peer relationships remains a persistent challenge [207].

Yet social communication deficits do not encompass the full clinical picture. The second domain of restricted, repetitive behaviors, interests, and activities coexists with them. Individuals with ASD commonly display rigid behavior patterns, strong adherence to familiar routines, unusually narrow interests, and a broad range of sensory abnormalities. Repetitive motor and verbal behaviors are among the most clinically conspicuous manifestations in this domain. These include repetitive hand flapping, body rocking, and spinning, as well as ritualized object manipulation such as lining up toys or repeatedly switching devices on and off. Speech patterns may also become repetitive, with immediate echolalia or stock phrases used out of context [208]. Insistence on sameness is equally prominent. Daily routines, environmental setups, and meal patterns are often followed with extreme rigidity. Even minor, unexpected changes can trigger intense distress, emotional outbursts, or behavioral dysregulation [209]. Interests also become remarkably narrow yet intensely focused. A person may become preoccupied with numbers, symbols, mechanical systems, or weather patterns to the exclusion of other activities [210]. Sensory abnormalities are emphasized in this domain as well. Individuals may show polarized responses to sensory input: indifference to pain, strong aversion to certain sounds or textures, or repetitive seeking behaviors such as sniffing or touching objects [45].

Taken together, these two symptom domains are not isolated. Social communication deficits isolate the individual from the social world, while repetitive behaviors and restricted interests limit flexibility and adaptation. Both begin early, shape development across the lifespan, and intertwine to produce the highly variable clinical presentation seen in ASD.

### Construction and application of classic autism animal models

To understand how autism develops, characterize behavioral changes, and develop new treatments, researchers need animal models that reliably recapitulate the core symptoms and pathology seen in patients. These models are the starting point for nearly all basic and translational work in the field.

Researchers currently rely mainly on *Drosophila*, mice, and rats. Each species has its own practical strengths in genetics, behavior, and biology. All of them can display core ASD features such as social deficits, repetitive behaviors, sensory abnormalities, and cognitive impairments [211]. They also share conserved genes, neurodevelopmental pathways, and neurotransmitter systems with humans [212].

*Drosophila* are especially useful because they are easy to handle, breed quickly, and have well-established genetic tools. These strengths make them ideal for large-scale screens and early studies of gene function [213]. Meanwhile, mice and rats have brain structures and physiology that more closely resemble those of humans. They can mirror the complex neural and behavioral changes seen in ASD, which is why they are the main models used to validate disease mechanisms and connect research to clinical work [12].

#### Behavioral characteristics and application value of* Drosophila* models

*Drosophila* are the most widely used invertebrate model in autism research. Their nervous system is much simpler than that of mammals, yet they carry many genes that are homologous to human ASD risk genes. Their neural development, neurotransmitter pathways, and basic behavior patterns are also highly conserved. As a result, *Drosophila* can reliably recapitulate several core ASD-related behaviors. They are valuable for exploring genetic mechanisms, studying behavioral regulation, and screening potential targets at scale [214]. In this model, abnormal social behavior is the most studied phenotype. Researchers have developed several well-established tests to measure it. Food competition assays reveal how *Drosophila* interact and cooperate in groups, reflecting basic social engagement. Courtship tests track behaviors such as following, tapping, wing vibration, and licking. These measure social recognition and mating-related social function [215]. Social space tests place *Drosophila* in an open arena and measure the distance they keep from one another. This provides a clear indicator of social approach or avoidance [216].

Beyond social deficits, *Drosophila* can also model other core and associated features of ASD. Grooming assays count self-cleaning episodes over a set period. This quantifies stereotyped, repetitive behavior and closely matches the repetitive and fixated behaviors seen in human ASD. Researchers have also developed a set of complementary tests. Negative geotaxis tests assess gravity response and motor coordination [217]. Feeding assays detect abnormal eating patterns that reflect the restricted eating habits seen in patients. Visual startle tests use sudden light flashes to measure shock response and habituation, reflecting sensory sensitivity [218]. Olfactory tests probe smell recognition and processing, helping explain deficits in smell processing [95]. Sleep assays track daily activity and rest cycles under controlled light–dark conditions, modeling the sleep disturbances common in ASD [219]. Learning and memory tests evaluate cognitive function through conditioning [220]. Open-field edge-preference tests measure anxiety-like behavior [221].

At the biological level, *Drosophila* and humans share highly conserved mechanisms related to ASD pathology. Both depend on core neurotransmitters such as GABA to maintain neural development and behavioral balance. When these transmitter levels become abnormal, they can directly lead to ASD-like social and repetitive phenotypes. This conservation across species offers a strong basis for studying disease mechanisms [222]. At the genetic level, many human ASD risk genes have clear homologs in *Drosophila*. For instance, the *Drosophila* genes *Nrx-1* and *Ssdp* correspond to human *NRXN1* and *SSBP3.* They similarly control neural development, synapse formation, and social behavior. These homologous genes provide a simple and efficient starting point for understanding the genetic mechanisms of human ASD [223, 224]. *Drosophila* models are also useful for studying how genes and environment interact. Researchers can easily adjust environmental factors such as temperature, diet, or sensory exposure. They can then combine these changes with specific genetic variants to see how the two interact to affect behavior and neural development. This approach aligns with current research in mammals, making *Drosophila* an important tool for exploring the complex causes of ASD [225].

Even so, *Drosophila* models have their limits. Their genes and neural circuits are far simpler than those of humans. They can show basic social behaviors, repetitive actions, sensory abnormalities, and mild learning deficits. The behavioral abnormalities induced by autism-associated gene mutations in *Drosophila* models are systematically summarized in Table 4. Yet they cannot recapitulate the higher-level social thinking, language, and complex reasoning seen in human autism. This gap makes it difficult to translate findings from *Drosophila* directly to the full behavioral picture seen in patients.

**Table 4:** Behavioral abnormalities observed in *Drosophila* models of autism-associated gene mutations

Autism-related gene	Food competition test	Courtship behavior test	Social space behavior test	Motor ability test	Sleep behavior test	Olfaction test	Learning and memory test	Repetitive behavior test	References
*Neuroligin3*	↑		↓	↓		↓			[217]
*KDM5*			↓			↓			[95]
*Aralar*	↓	↓	↓					↓	[226]
*KMT2C*		↓	↓						[227]
*UBE3A*				↓	↓				[104]
*SHANK*				↓		↓	↓		[228]
*MECP2*			↓	↓					[229]
*FMR1*			↓		↓		↓	↓	[230]

*KDM5* Lysine Demethylase 5, *KMT2C* Lysine Methyltransferase 2 C, *UBE3A* Ubiquitin Protein Ligase E3A, *SHANK* SH3 and Multiple Ankyrin Repeat Domains, *MECP2* Methyl-CpG Binding Protein 2, *FMR1* Fragile X Messenger Ribonucleoprotein 1

#### Behavioral phenotypes and applicability of classic mammalian models

Rodents (chiefly mice and rats) currently dominate ASD preclinical work. Their neural architecture, immunological makeup, and neurotransmitter systems share sufficient conservation with humans to support the modeling of core ASD symptoms and common comorbidities [105]. Yet this conservation is partial. Rodents lack the higher-order social cognition, language circuitry, and prefrontal complexity that characterize human autism, so behavioral readouts in these animals inevitably represent simplified approximations rather than true equivalents [231].

For social impairment, the central diagnostic domain, researchers rely on the three-chamber social test, social preference test, and peer interaction test to index social avoidance, diminished reciprocity, and communication deficits [232]. These tasks capture basic social motivation and olfactory-driven affiliation, but they cannot approximate the nuanced pragmatics of human language or theory-of-mind impairments. Repetitive motor patterns, excessive grooming, circling, head shaking, and rigid patterns of environmental exploration, are also readily observable and arguably more clinically grounded than the grooming stereotypies described in *Drosophila* [233]. Sensory sensitivity is indexed through acoustic, visual, or nociceptive challenge. Sleep fragmentation is tracked via monitoring systems [234, 235]. Cognitive deficits are evaluated with water maze and novel object recognition tests [236]. Collectively, this battery allows investigators to parse how genetic or environmental insults shape not only core features but also the peripheral conditions that frequently accompany them.

The value of rodent models extends well beyond behavior. They permit invasive interrogation of synaptic pathology, intracellular signaling cascades, neuroimmune inflammation, and excitation-inhibition imbalance at levels impossible in human subjects [237]. Nevertheless, this strength comes with a persistent weakness: preclinical findings in rodents often fail to translate to human trials. Differences in brain development timing, immune priming, and pharmacokinetics contribute to high attrition rates, reminding the field that a mechanistic observation in the mouse is a starting hypothesis, not a proven clinical target.

Consequently, most laboratories now adopt a tiered workflow rather than relying on any single species. Invertebrate models (fast, cheap, and genetically tractable) handle initial screening and target identification. Rodents then assume the heavier burden of circuit-level validation, immune profiling, and drug-efficacy testing. This sequential pipeline mitigates the inherent constraints of each system and provides a more rigorous foundation for understanding ASD heterogeneity and refining therapeutic candidates [213].

#### Characteristics and applicability of non-classical animal models

ASD research extends beyond *Drosophila* and rodents. Zebrafish, *C. elegans*, and non-human primates each fill gaps left by classic models: zebrafish permit real-time developmental observation, C. elegans isolates basic mechanisms for study, and non-human primates more closely approximate human patients. Together, they form a complete research chain from basic science to clinical application.

Zebrafish are the fastest-growing alternative. Their genome is over 87% identical to humans, with highly conserved ASD risk genes such as *SHANK3*, *NRXN1*, and *AUTS2* [238, 239]. Neural development, synapse formation, and brain regionalization are also remarkably similar. Their transparent, externally developing embryos allow real-time tracking of neural proliferation, migration, differentiation, and synapse formation without dissection, ideal for studying early neurodevelopmental injury [240]. Behaviorally, shoal preference and peer interaction tests index social deficits; circling, fixed-path swimming, and repeated wall-touching quantify repetitive behavior; acoustic, visual, and tactile stimuli probe sensory hypersensitivity; light–dark switching assesses anxiety; and shuttle learning and place memory tests measure cognitive impairment [241]. They are also practical: efficient breeding, low cost, and easy embryo drug delivery suit them for environmental toxin studies, developmental drug testing, and large-scale small-molecule screening [242]. Their simpler nervous system cannot, however, reproduce higher-order social cognition or complex emotional abnormalities [243].

*C. elegans* sits at the opposite extreme. As the simplest multicellular model, it offers a transparent body, short lifespan, fully mapped genetics, and rapid, large-scale testing. It is rarely used for routine ASD behavioral studies; instead, it tests single-gene function and basic neural control mechanisms [244]. Despite its simplicity, it retains core human pathways including synaptic transmission, GABA signaling, and neuronal apoptosis. Basic locomotion, chemotaxis, food sensing, and synaptic transmission efficiency serve as indirect readouts of ASD-related neural dysfunction [245, 246]. Its main value lies in large-scale screening and isolating individual mechanisms, pinpointing how single genes or pathways contribute to disease and providing starting points for work in *Drosophila* and rodents [247]. Yet its narrow behavioral repertoire and lack of complex neural circuits restrict it to exploratory screening; it cannot support phenotypic validation or in-depth mechanism analysis [248].

Non-human primates closely match humans in brain structure, neural circuits, higher cognition, and social systems. They reproduce high-level social deficits, language impairments, complex emotional abnormalities, and rigid cognitive behaviors that rodents cannot capture, making them the closest available model of human patients [249]. Their ASD susceptibility gene sequences and expression patterns are almost identical to humans [250]. Gene-edited monkeys with ASD-related mutations display autism-like behaviors such as social avoidance, low social motivation, repetitive movements, difficulty adjusting to new environments, and cognitive inflexibility, and replicate complex human pathologies including abnormal cortical development, disrupted synaptic plasticity, neuroimmune inflammation, and abnormal brain network connectivity [251]. Their greatest advantage is modeling complex human social and cognitive behavior for detailed mechanism analysis, therapeutic target validation, long-term drug safety testing, and gene therapy translation [252]. These strengths are offset by long breeding cycles, high costs, limited sample sizes, and difficult genetic editing, which preclude large-scale screening or early exploration. They are reserved instead for validating key mechanisms and bridging basic research with clinical therapy [253, 254].

**Fig. 4:**
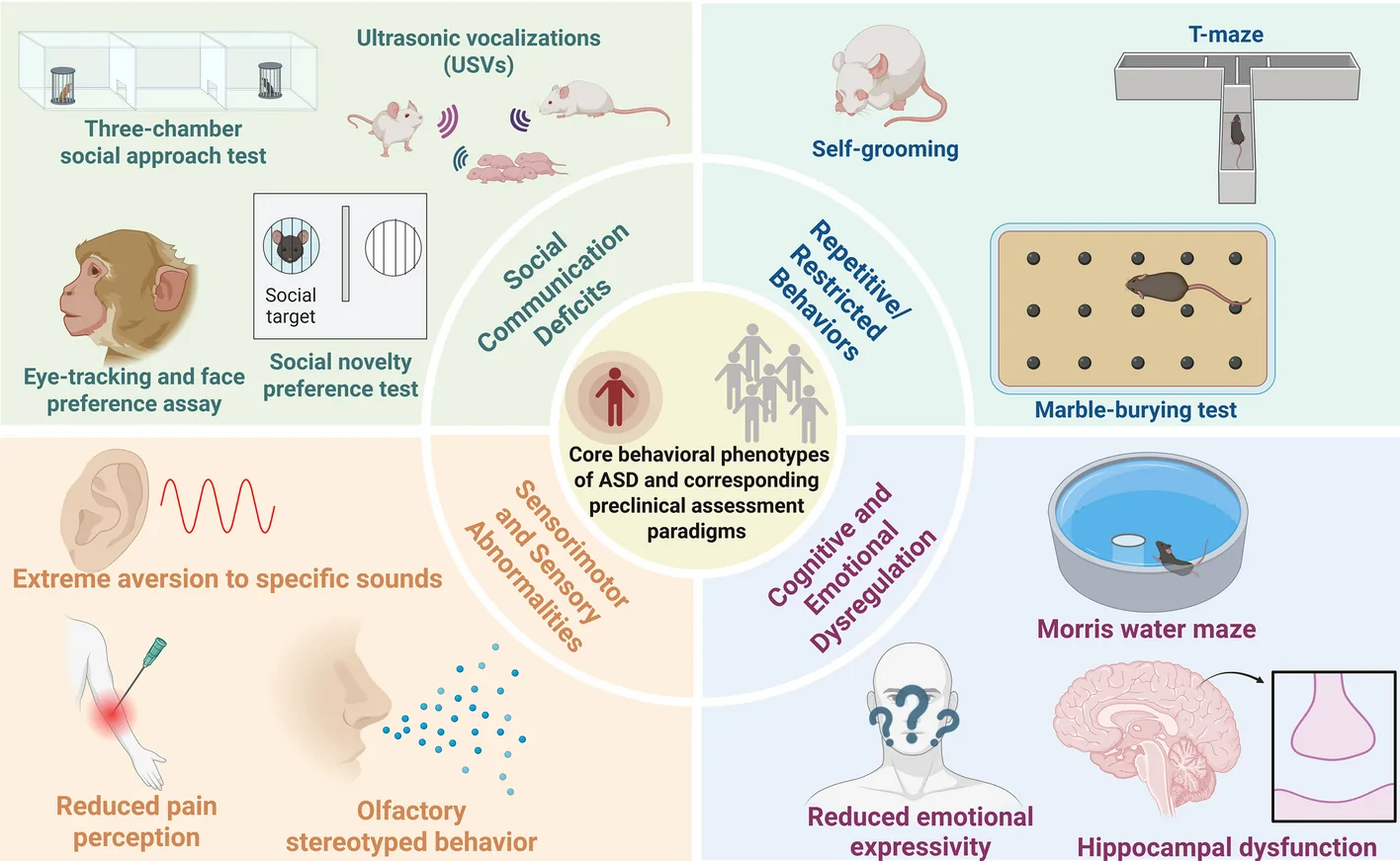
Preclinical behavioral assays for assessing core ASD-related phenotypes across model organisms. Core behavioral phenotypes of ASD and corresponding animal behavioral assays. The core symptoms of ASD are categorized into four dimensions: Social dimension, Repetitive or stereotyped dimension, Sensorimotor dimension, and Emotional-cognitive dimension. Representative assays for each dimension include: (1) *Social* — Three-chamber social approach test, Ultrasonic vocalizations (USVs), Social novelty preference test, and Eye-tracking/face preference assay in non-human primates; (2) *Repetitive/Stereotyped* — Self-grooming, T-maze, and Marble-burying test; (3) *Sensorimotor* — Tactile-sensitivity test, Prepulse inhibition, and Rotarod test; and (4) *Emotional-Cognitive* — Morris water maze and Social transmission of taste avoidance in non-human primates. These paradigms are widely used in rodent and non-human primate models to systematically evaluate ASD-relevant core phenotypes. Created in BioRender.com. USVs Ultrasonic vocalizations

Together, these three models occupy distinct, complementary roles: *C. elegans* for initial gene and pathway exploration, zebrafish for early neural development and large-scale drug screening, and non-human primates for complex mechanisms and clinical translation. In practice, researchers work in tiers: *C. elegans* or *Drosophila* for initial screening, zebrafish for developmental validation, mice or rats for in-depth mechanism analysis, and primates for final clinical translation. This tiered approach reduces single-species bias, improving mechanistic depth and translational efficiency (Fig. 4).

## Management, intervention and research prospects

AI, multi-omics, and other advanced technologies are pushing ASD diagnosis to become earlier, more precise, and more objective. As biological subtypes become clearer, clinical care is also shifting away from broad, standardized approaches toward treatment tailored to specific subtypes and individual needs.

An integrated intervention system is now in place. Behavioral and educational interventions sit at the center, medical treatments for specific symptoms provide additional support, and lifelong services ensure ongoing care. In combination, these components relieve core symptoms, improve social adaptability, and reduce the burden on families and society.

### Management

#### Educational and behavioral intervention

When clinicians can identify risk earlier and more accurately, the opportunity for early intervention expands. Education and behavioral intervention remain the first-line approach in ASD care, supported by the most robust evidence. They are the most effective means to improve social communication and reduce repetitive behaviors, using neurodevelopmental principles and behavioral techniques to develop functional skills.

In practice, naturalistic developmental interventions are readily integrated into family and school routines, rendering them particularly suitable for young children with milder symptoms requiring comprehensive training. When combined with communication aids, they can also markedly improve social expression in children with language delays. Classic methods such as structured teaching and applied behavior analysis are well validated and can be tailored to developmental stage and symptom severity. Artificial intelligence is now enhancing this field by collecting and analyzing daily behavior in real time, optimizing both clinical and home-based intervention plans, and addressing disparities in local rehabilitation resource availability.

As biological subtype research moves forward, matching rehabilitation training to subtype traits has become an important way to enhance both precision and functional outcomes. Nevertheless, current programs still face challenges in maintaining long-term gains and in transferring training outcomes to daily functioning. Moving forward, researchers will need to integrate multi-omics data with intelligent monitoring to continuously refine individualized, flexible intervention plans [255–261].

#### Clinical medical treatment

Behavioral intervention restores functional capacity, but patients often need medical support to stabilize symptoms such as anxiety, hyperactivity, emotional dysregulation, and sleep problems. Importantly, no pharmacological agent currently ameliorates the core social deficits or repetitive behaviors of ASD. Current pharmacological intervention focuses on symptom alleviation and standardized comorbidity management, optimizing associated conditions so that educational and behavioral intervention can proceed on a more stable foundation. Because individuals exhibit markedly variable responses to these drugs, adverse effects require careful monitoring during long-term use.

Individuals with ASD often have other medical conditions, and the type of comorbidity is closely associated with biological subtype. If left untreated, these comorbid conditions can exacerbate core symptoms. Therefore, clinical practice requires team-based care, with standardized symptom management matched to the specific comorbid condition. Beyond current symptom drugs, researchers are gradually exploring new directions such as metabolic pathway modulation, gene-targeted intervention, and gut microbiome regulation. However, most of these new approaches remain in basic research or early clinical testing, and comprehensive safety and efficacy evaluations are still needed before they can be widely used [262–265].

#### Supportive services and lifelong care

Drugs and behavioral interventions can improve symptoms, but they cannot replace the continuous support patients need in family, school, and community life. Supportive services constitute the integrative framework. They address the deficiencies in medical and educational care and facilitate patient integration into daily routines. This support operates across three levels. At the family level, the focus encompasses caregiver training, counseling, and resource coordination. This alleviates caregiver burden and enhances the quality of long-term home care. At the school level, inclusive educational environments should be established, with individualized educational plans tailored to the patient's developmental level, complemented by public awareness initiatives to create a supportive and equitable environment. At the community level, rehabilitation infrastructure requires continued expansion, with telemedicine mitigating geographic barriers and public awareness campaigns progressively reducing stigma [266–270].

Most importantly, this support system must encompass the full lifespan, from childhood through adolescence to adulthood. For adult patients, the focus encompasses job skills training and independent living guidance, addressing stage-specific needs and advancing social support services toward greater diversity, reach, and personalization.

## Research prospects and future direction

ASD diagnosis currently relies mainly on DSM-5-TR and ICD-11 criteria, with behavioral assessment as the core tool. This approach often leads to delayed diagnosis, missed cases, and a lack of specific biological markers [271].

These gaps are pushing researchers to pursue new approaches on two fronts. In behavior and imaging, artificial intelligence is already showing promising results. Home-video analysis reaches Receiver Operating Characteristic (ROC) values of 0.83; neuroimaging achieves 80% to 85% accuracy; and multimodal models may push precision even higher. Still, most of these tools remain in clinical validation. Molecular tools are also emerging. Polygenic risk scores can stratify patients by risk level; blood transcriptome signatures allow non-invasive early screening; metabolomics has shown that branched-chain amino acid dysregulation and mitochondrial dysfunction are common in ASD; and gut microbiome changes can serve as ancillary clues [189, 272, 273]. The real challenge now is how to translate these lab findings into practical clinical tests. If researchers can combine these molecular markers and integrate them with behavioral assessments, a more objective diagnostic model could emerge.

Better diagnosis also needs to connect to subtyping and therapeutic pathways. The four stable biological subtypes reported in *Nature Genetics* in 2025 provide robust evidence for precision classification [256]. The next objective is to combine clinical signs, genetic backgrounds, and multi-omics data into a single classification system, while building a tiered network that links community screening, regional diagnosis, and team-based intervention. Telemedicine can facilitate equitable resource distribution and enhance early screening at the primary care level, thereby actualizing the principle of "early detection, early classification, and early intervention". Integrating advanced technologies and molecular diagnostics into current diagnostic systems is the best way to overcome existing limitations and improve the quality of early identification and precise classification. This also establishes the foundation for standardized intervention.

As support systems progressively encompass the full lifespan, ASD research as a whole is moving from basic mechanistic research to precision diagnosis and treatment. The remaining challenges are the marked disease heterogeneity and the challenges in translating biological markers into routine clinical practice. Future research must continue to explore genetic diversity and gene-environment interactions in disease pathogenesis, bridge the gap between precise subtyping and clinical intervention, standardize care delivery, and balance diagnostic and rehabilitation resources across regions. Through robust interdisciplinary collaboration and sustained technological innovation, the field can continuously improve the comprehensive care continuum, early screening, precise diagnosis, layered intervention, and long-term support, to enhance the overall quality of ASD care and substantially improve long-term quality of life for patients [274].

## Conclusion and prospects

ASD is a highly heterogeneous neurodevelopmental disorder arising from dynamic interactions between genetic predisposition and environmental risk factors during critical developmental periods. Cumulative evidence indicates that multilevel perturbations spanning synaptic structure, intracellular signaling cascades, immune homeostasis and neural circuit dynamics collectively drive the diverse behavioral phenotypes of ASD. This review synthesizes current progress in ASD epidemiology, genetic pathogenesis, preclinical modeling, and clinical interventions, and further highlights key limitations and future translational priorities in this field. It is worth distinguishing our approach from existing comprehensive reviews. Whereas landmark syntheses have typically excelled within single dimensions, whether mapping genetic architecture or outlining clinical care systems, our review integrates these scales through the lens of mechanistic convergence. By using E/I imbalance as the central integrative hub, we trace a continuous path from population-level epidemiological risk factors, through molecular and circuit-level pathophysiology, to mechanism-informed intervention design. This perspective, we believe, offers a distinctive contribution to the current ASD review literature.

Recent epidemiological data from the GBD 2021 and WHO demonstrate a substantial global ASD disease burden, with pronounced regional disparities in reported prevalence. Such variations largely reflect differences in diagnostic accessibility, public awareness and screening coverage rather than true biological incidence. Notably, China exhibits considerable underdetection, particularly among females and individuals with subtler phenotypic presentations, emphasizing the urgent need for equitable, lifespan-oriented public health screening systems to reduce diagnostic inequality.

Genetic studies have confirmed ASD as a highly heritable disorder governed by a combination of high-impact rare variants, copy number variations, and polygenic susceptibility. Core ASD-risk genes converge on neurodevelopmental processes encompassing synaptic formation, neuronal migration, and activity-dependent chromatin remodeling, through canonical signaling pathways such as mTOR, Ras-ERK-MAPK, Wnt/β-catenin, and Notch. Despite these advances, current genetic and pathway research has notable limitations. Most identified risk variants are population-specific and fail to fully explain ASD heterogeneity, leaving a large proportion of sporadic cases genetically unexplained. Existing pathway analyses largely focus on isolated cascades, lacking systematic characterization of pathway crosstalk and compensatory mechanisms. Furthermore, conventional bulk genomic data cannot resolve cell-type-specific or dynamic spatiotemporal pathogenic changes during neurodevelopment, and the functional validation of most candidate genes and pathways remains insufficient to support clinical translation.

Genetic vulnerabilities interact with immune dysregulation and environmental cues to trigger multilayered brain pathology in ASD. Microglial overactivation and peripheral immune disturbance exacerbate neuronal impairment, while disrupted glutamatergic-GABAergic balance represents a convergent pathological endpoint underlying core autistic behaviors. Epigenetic modifications further modulate gene penetrance and individual heterogeneity. Although research has shifted from single-gene causality to multidimensional network dysfunction, integrating these layered mechanisms into a unified pathogenic framework remains a major challenge.

Animal models serve as essential translational tools for dissecting ASD mechanisms, with complementary model systems spanning *Drosophila*, zebrafish, rodents, and non-human primates offering distinct experimental advantages. Nevertheless, significant translational gaps persist. Preclinical behavioral assays cannot fully recapitulate human social communication and higher-order cognitive deficits, contributing to the high failure rate of preclinical therapeutic candidates in clinical trials. Standardized cross-species phenotyping and harmonized translational biomarkers are therefore required to improve research reproducibility and clinical relevance.

Clinically, ASD diagnosis still relies solely on behavioral criteria, lacking validated molecular biomarkers for routine screening. Widespread diagnostic delay prevents many children from accessing optimal early intervention. Current treatments are limited to behavioral rehabilitation and symptomatic management of comorbidities, with no approved therapies targeting core social and repetitive symptoms. While emerging molecular subtyping provides new opportunities for precision medicine, integrating biological signatures with clinical phenotypes to establish operational diagnostic frameworks remains an unmet clinical need.

To bridge these persistent translational gaps, coordinated advances across basic research, clinical diagnosis, targeted therapy and long-term social support are essential. Mechanistically, integrating prospective birth cohorts with single-cell sequencing, spatial transcriptomics and brain organoid models can systematically map how genetic variants disrupt neurodevelopment across biological scales. Combined multi-omics profiling and CRISPR-based epigenome editing will further clarify gene-environment crosstalk and refine integrated pathogenic networks. Clinically, low-cost, non-invasive molecular biomarkers, including blood transcriptomic and metabolic signatures, hold promise for community-wide early screening. A telemedicine-supported multi-tiered healthcare system linking community screening, specialist diagnosis and multidisciplinary intervention can effectively alleviate socioeconomic and geographic healthcare disparities.

In therapeutic development, the traditional one-size-fits-all model should be replaced by mechanism-based, subgroup-tailored interventions, requiring rigorous clinical validation with standardized outcome measures and long-term follow-up to confirm treatment durability and generalizability. Equally critical is the improvement of lifespan support systems. Current services for adolescents and adult patients remain fragmented, whereas ASD-associated care demands persist across the entire lifespan. Strengthening caregiver training, inclusive education, vocational rehabilitation and community healthcare, together with public awareness initiatives to reduce social stigma, is essential for improving long-term quality of life for individuals with ASD.

In summary, ASD represents a genetically and clinically heterogeneous neurodevelopmental disorder with multilayered pathogenic perturbations. Despite substantial progress in etiological exploration, the field continues to face a major translational bottleneck in translating basic biological discoveries into clinically actionable tools. Future advances depend on the systematic integration of mechanistic research, clinical innovation and public health infrastructure. Such interdisciplinary efforts will facilitate early detection, precise subtyping and individualized intervention, ultimately improving long-term patient outcomes and reducing the socioeconomic burden of ASD on families and healthcare systems.

## Data Availability

Not applicable.
